# The Nutri-Score algorithm: Evaluation of its validation process

**DOI:** 10.3389/fnut.2022.974003

**Published:** 2022-08-15

**Authors:** Daphne L. M. van der Bend, Manon van Eijsden, Michelle H. I. van Roost, Kees de Graaf, Annet J. C. Roodenburg

**Affiliations:** ^1^Marketing and Consumer Behaviour Group, Wageningen University, Wageningen, Netherlands; ^2^Voedingsjungle, Amersfoort, Netherlands; ^3^Division of Human Nutrition, Wageningen University, Wageningen, Netherlands; ^4^Department of Food and Industry, HAS University of Applied Sciences, ‘s-Hertogenbosch, Netherlands

**Keywords:** Nutri-Score, nutrient profile models, front-of-pack labeling, validity, review

## Abstract

The Nutri-Score front-of-pack label, which classifies the nutritional quality of products in one of 5 classes (A to E), is one of the main candidates for standardized front-of-pack labeling in the EU. The algorithm underpinning the Nutri-Score label is derived from the Food Standard Agency (FSA) nutrient profile model, originally a binary model developed to regulate the marketing of foods to children in the UK. This review describes the development and validation process of the Nutri-Score algorithm. While the Nutri-Score label is one of the most studied front-of-pack labels in the EU, its validity and applicability in the European context is still undetermined. For several European countries, content validity (i.e., ability to rank foods according to healthfulness) has been evaluated. Studies showed Nutri-Score's ability to classify foods across the board of the total food supply, but did not show the actual healthfulness of products within different classes. Convergent validity (i.e., ability to categorize products in a similar way as other systems such as dietary guidelines) was assessed with the French dietary guidelines; further adaptations of the Nutri-Score algorithm seem needed to ensure alignment with food-based dietary guidelines across the EU. Predictive validity (i.e., ability to predict disease risk when applied to population dietary data) could be re-assessed after adaptations are made to the algorithm. Currently, seven countries have implemented or aim to implement Nutri-Score. These countries appointed an international scientific committee to evaluate Nutri-Score, its underlying algorithm and its applicability in a European context. With this review, we hope to contribute to the scientific and political discussions with respect to nutrition labeling in the EU.

## Introduction

**Figure 1:**
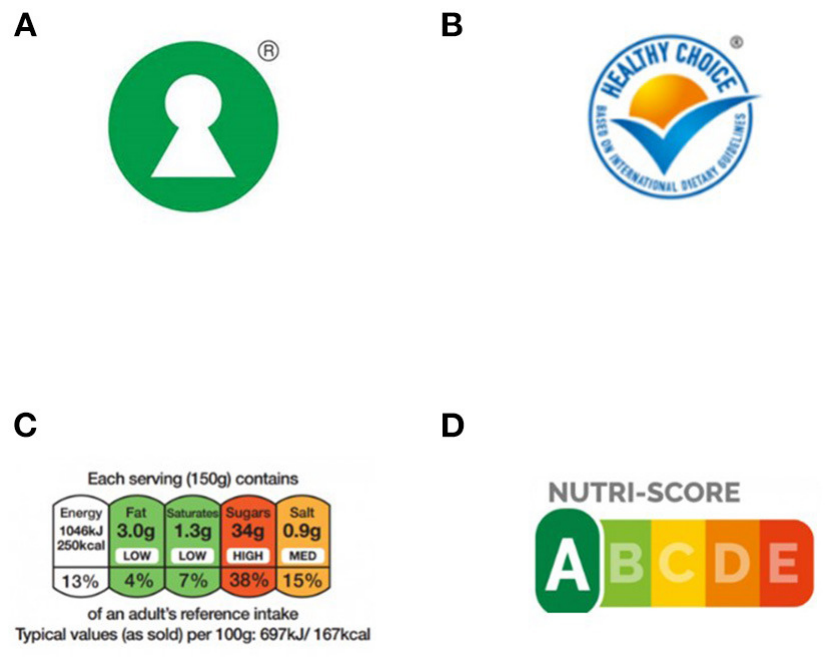
The four main FOP labels currently in use in Europe: Keyhole **(A)**, choices **(B)**, multiple traffic light **(C)**, and Nutri-Score **(D)**.

In recent years, nutrition labeling has gained increasing attention, both in scientific and political discourse. Especially front-of-pack (FOP) labeling, as a WHO recommended policy tool to promote healthier diets and prevent non-communicable diseases (1), has sparked discussion in the European Union's political arena (2). While the mandatory elements of nutrition labeling–usually presented in a back-of-pack nutrition declaration table–are laid down in the EU regulation No 1169/2011 on the provision of food information, FOP labeling is as yet still voluntary with various forms allowed, as long as these comply with the criteria set out in the Regulation (3). Consequently, a variety of schemes are currently in use in the EU member states and the UK, varying in visual presentation, type of message (informative or directive) and focus (overall nutrition quality or nutrient-specific) (3, 4). The most well-known among these schemes are the Keyhole logo (5), the Choices logo (6), the Multiple Traffic Light (MTL) scheme (7) and the more recently developed Nutri-Score label (8) (Figure 1).

Keyhole, Choices and Nutri-Score are all directive, interpretative labels, i.e., labels that summarize the healthiness of the products without displaying nutritional information. The MTL scheme may be considered semi-directive, as it combines nutrient information with interpretive color coding using the familiar traffic light colors of red, orange and green. In a similar visual expression, the Nutri-Score label uses both colors and letters from A to E to rank the nutritional quality of products, both across and within food groups. The Keyhole logo and the Choices logo, on the other hand, use their visuals only to point out the healthier food products within a food group or food category (9).

At the moment, Nutri-Score has been implemented not only in France–from where it originates–but also in Belgium. Moreover, implementation has been announced in Spain, Germany, Switzerland, Luxembourg and the Netherlands (10). In the Netherlands, implementation was made conditional on adapting the algorithm underpinning the label, to ensure alignment with the national dietary guidelines (11). Indeed, examining the adherence to national dietary guidelines is a relevant measure of convergent validity and has been recommended by the WHO as one of the essential steps before implementing a FOP label in their “Guiding principles and framework manual for FOP labeling for promoting healthy diet” (12). More specifically, the WHO outlines three essential steps to be taken to validate the nutrient profile model underlying any proposed FOP label:

1) to examine content validity–does the algorithm allow the categorization of foods and beverages according to healthfulness;
2) to examine convergent validity–does the categorization of products using the algorithm compare to the categorization of products using another system (e.g., the national dietary guidelines);
3) to examine predictive validity–if the algorithm is applied to population dietary data to indicate the healthfulness of the diet, what prospective associations are observed in terms of disease risk?

This review aims to evaluate these three critical validation steps of the Nutri-Score label. It describes the adaptations made to the original FSA Ofcom nutrient profile model–which was designed for a different purpose, i.e., to regulate the marketing of foods to children–to arrive at the Nutri-Score model as introduced in France and the validity studies that were performed. Although studies on Nutri-Score's validity were initially performed only in the French context, studies within and across other European countries have been conducted with increasing frequency. These studies mostly focused on content and predictive validity. While this review sets out to provide a comprehensive overview of these French and European studies, it cannot be considered exhaustive as it includes only papers in the English language. Papers were derived from the French government's overview of the Nutri-Score validation process (13, 14). To check for potentially missing papers, an additional PubMed Search was executed. Supplementary material 1 provides the search strategy and an overview of the studies included.

## Nutri-Score's basis: The food standards agency nutrient profile model

The algorithm underpinning the Nutri-Score label is derived from the UK Food Standards Agency (FSA)/Office of Communication (Ofcom) nutrient profile model, also known as “model WXYfm.” This model was developed as a tool to regulate the marketing of foods to children in the UK and has been applied since 2007. Development and validation of the model was comprehensively reviewed by Rayner (15). The development included three stages. In the first stage, it was agreed that the model should use a scoring system and include nutrients and other food components that should be encouraged, as well as nutrients and food components that should be discouraged. In the second stage, a prototype model was discussed with a range of stakeholders, and in the last stage the final model was agreed upon in consultation with the UK Government Scientific Advisory Committee on Nutrition (15).

The final FSA/Ofcom model provides a single score (hereafter referred to as the FSA-score) for any given food product based on calculating the number of points for “negative” nutrients which can be offset by points for “positive” nutrients (see Box 1). Points are allocated on the basis of the nutritional content in 100 g of a food or drink. Foods and beverages are scored similarly, or “across-the-board,” i.e., the same set of criteria are used for all products, however the cut-offs used to determine whether the products may be marketed to children differs between foods and beverages (see Supplementary material 2 as well) (16).

Box 1 **The FSA/Ofcom algorithm**

The FSA/Ofcom model uses an algorithm to calculate a score for the nutritional quality of a food. Based on that score, here referred to as the FSA-score, foods and beverages are classified into one of two groups: not allowed to market to children or allowed to market to children

The algorithm is as follows:

1) For each food and beverage, negative points are calculated, based on their nutritional composition of ‘negative' nutrients per 100 g. Note: point allocation is positive, so the more energy, saturated fat, sugar and sodium a product contains, the higher the number of negative points:

**Table:** 

**Negative points**	**Energy (kJ)**	**Saturated fat (g)**	**Total sugar (g)**	**Sodium (mg)**
0	≤ 335	≤ 1	≤ 4.5	≤ 90
1	>335	>1	>4.5	>90
2	>670	>2	>9	>180
3	>1,005	>3	>13.5	>270
4	>1,340	>4	>18	>360
5	>1,675	>5	>22.5	>450
6	>2,010	>6	>27	>540
7	>2,345	>7	>31	>630
8	>2,680	>8	>36	>720
9	>3,015	>9	>40	>810
10	>3,350	>10	>45	>900

*Total negative points = (points for energy) + (points for saturated fat) + (points for total sugar) + (points for sodium).*

2) For each food and beverage positive points are calculated, based on their nutritional composition of “positive” nutrients per 100 g:

**Table:** 

**Positive points**	**Fruit, vegetables, legumes and nuts (%)**	**Fiber (non-starch polysaccharides) (g)**	**Protein (g)**
0	≤ 40	≤ 0.7	≤ 1.6
1	>40	>0.7	>1.6
2	>60	>1.4	>3.2
3	-	>2.1	>4.8
4	-	>2.8	>6.4
5	>80	>3.5	>8.0

*Total positive points = [points for fruits, vegetables, legumes, and nuts (FVLN)] + (points for fiber) + (points for protein).*

3) To compute the FSA-score the N-points and P-points are balanced according to the following formula:

^*^ if negative points <11; FSA-score = negative points–positive points

^*^ if negative points ≥ 11 & points FVLN = 5; FSA-score = negative points–positive points

^*^ if negative points ≥ 11 & points FVLN <5; FSA-score = negative points–(points FVLN + points fiber)

The resulting FSA-score gives an indication of the nutritional quality of a product, with a lower score indicating a higher nutritional quality. For the purpose of the FSA/Ofcom model, i.e., advertising control, the following cut-offs were used:

Foods: ≥4–“less healthy,” no marketing; <4–marketing allowed;

Beverages: ≥1–“less healthy,” no marketing; <1–marketing allowed.

*Example: cottage cheese*

**Table:** 

**Nutrient**	**Per 100 g**	**Negative points**	**Positive points**
Energy	381 kJ	1	
Saturated fat	2.2 g	2	
Sugar	2.8 g	0	
Sodium	300 mg	3	
Fruit, vegetables, legumes and nuts	0 %		0
Fiber	0 g		0
Protein	12 g		5
**Total**		**6**	**5**

*Total points = 6 – 5 = 1. This cottage cheese would therefore not be subject to marketing restrictions.*

The FSA/Ofcom model was shown to have good agreement between the ranking of products by the model and the ranking of products by nutritionists (Spearman's ρ 0.79) (17), and good agreement between the model and the UK's national food guide for the classification of products in healthier or less healthy (k 0.69) (18), both measures of convergent validity.

## Development and validation process of the Nutri-Score model

**Figure 2:**
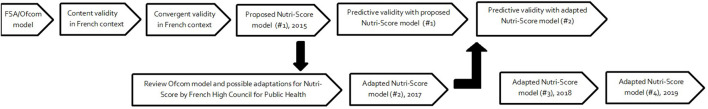
The development and validation process for the Nutri-Score model prior to and during implementation in France.

In 2014, the first paper on the development of the Nutri-Score label was published. This paper describes the adaptation of the categorization: from binary to five categories (hereafter referred to as Nutri-Score classes). It was assumed that a multicategory label would prevent dichotomous thinking in “bad” and “good” foods and entice manufacturers to product reformulation (19). After assessing content validity for this categorization (19–21), convergent validity was assessed by comparing the categorization of food and beverages according to the model with the French nutritional recommendations (21). Based on these results, a subsequent adaptation in the algorithm of the model was proposed (here referred to as Nutri-Score model #1, Figure 2). This proposal was further adapted by the French High Council for Public Health to establish the final algorithm underlying the Nutri-Score label that was implemented in France in 2017 (here referred to as Nutri-Score model #2, Figure 2) (22). Multiple predictive validity studies, assessing the association of both the proposed Nutri-Score model (#1) and the adapted Nutri-Score model (#2) with disease risk in the French population were subsequently performed (23–29).

In the meantime, the Nutri-Score algorithm was further adapted to use the Association of Official Analytical Chemists (AOAC) calculation rather that the non-starch polysaccharide measurement for fiber (model #3, Figure 2), as the aforementioned method was set as the reference method in 2018 by the French Ministry of Health (personal communication Dr. Julia). Also, in the calculation used for liquids, the reference unit became 100 ml rather than 100 g, in line with the nutrition declaration on the package to ensure transparency for consumers. The most recent adaptation of the model happened in October 2019 (model #4, Figure 2). To better take the nutritional recommendations for oils in Europe into account, the computation of the content for the food group fruit, vegetables, legumes and nuts (FVLN) was adapted to also include the content of rapeseed oil, walnut oil and olive oil (see also Supplementary material 2) (30). Figure 2 presents an overview of the development and validation process for the Nutri-Score model. Below, we first describe the three main validation steps in the French context in detail, after which we elaborate on the validation of the Nutri-Score in the European context.

## Nutri-Score's content validity in the French context: Classification of foods

In the context of a nutrient profile model, content validity refers to the ability of the model to classify products according to healthiness (12). For Nutri-Score, this was assessed in three subsequent studies, using the original FSA/Ofcom model (19–21). Using the quintile distribution of the FSA-score, 5 Nutri-Score classes were formed and this classification was compared with the food group classification of the French Programme National Nutrition Santé (PNNS) (19–21).

In two studies (20, 21), the ability to discriminate the nutritional quality of foods was estimated by the number of Nutri-Score classes in each food group (e.g., cereals, legumes and potatoes), each food category (e.g., breakfast cereals), and for similar products of different brands (e.g., mueslis). Tables 1, 2 give the distribution of the PNNS food groups across quintiles of the FSA-score distribution in the French NutriNet Santé food composition database (19) and in the Open Food Facts database (21), respectively. Table 3 shows the distribution of breakfast cereals, including the distribution of equivalent products of different brands (20).

**Table 1:** Comparison of non-weighted and weighted analysis as presented in Julia et al. (19): distribution (%)^a^ of food groups across quintiles of FSA-score distribution in the French NutriNet Santé food composition database (non-weighted *n* = 3,331; weighted *n* = 1,878).

**Food group**	**Q1 (< -2)**	**Q2 (-1;3)**	**Q3 (4;11)**	**Q4 (12;16)**	**Q5 (≥17)**
**Fruit and vegetables**
Unweighted	66.2	22.9	10.3	0.6	0.0
Weighted	82.4	15.4	2.2	0.0	0.0
**Cereals, legumes and potatoes**
Unweighted	29.3	20.4	30.5	15.1	4.7
Weighted	38.2	52.2	8.4	0.6	0.6
**Milk and dairy**
Unweighted	4.4	25.6	28.4	18.0	23.6
Weighted	12.2	64.9	5.6	5.2	12.0
**Meat, fish and eggs**
Unweighted	27.9	32.5	11.5	11.8	16.4
Weighted	33.3	34.1	11.3	8.1	13.2
**Sugary snacks**
Unweighted	1.1	3.0	17.1	31.5	47.2
Weighted	0.0	3.7	41.7	32.6	22.0
**Salty snacks**
Unweighted	15.5	16.2	31.1	18.2	18.9
Weighted	31.7	10.5	21.6	16.7	19.5
Fat and sauces
Unweighted	2.9	9.6	19.9	20.6	47.1
Weighted	2.1	3.8	5.6	21.7	66.6
**Composite foods**
Unweighted	19.1	34.7	20.7	18.9	6.6
Weighted	19.8	45.5	14.9	13.7	6.2

a *Percentages reported were rounded to the nearest decimal for the present review*.

**Table 2:** Shifts in distribution (%)^a^ **across scoring** categories for food groups for which the algorithm was adapted^b^ for better adherence to dietary guidelines, as described in Julia et al. (21).

	**Nutri-Score nutritional quality category**				
	**A**	**B**	**C**	**D**	**E**
**Foods**					
Category cut-offs	< -2	−1–3	4–11	12–16	≥17
Fruit and vegetables					
Original	72.1	23.3	4.3	0.4	-
Modified	71.3	21.1	6.6	0.8	0.3
Dried fruits					
Original	18.2	66.7	12.1	3.0	-
Modified	-	18.2	72.7	6.1	3.0
Milk and dairy					
Original	5.2	34.1	20.9	15.8	24.0
Modified	5.2	34.1	26.4	26.8	7.5
Cheese					
Original	-	3.5	1.2	22.0	73.3
Modified	-	3.5	21.2	62.0	13.3
Fats and sauces					
Original	2.2	15.6	19.1	24.9	38.2
Modified	2.2	16.1	33.8	31.2	16.7
Fats					
Original	-	0.5	2.1	22.2	75.1
Modified	-	1.6	38.1	37.6	22.8
Salty snacks					
Original	2.9	9.8	45.0	25.6	16.7
Modified	1.0	8.1	46.5	27.1	17.3
Nuts					
Original	15.5	29.3	50.0	5.2	-
Modified	-	15.5	62.1	17.2	5.2
Beverages					
**Category cut-offs**	<0	1–4	5–8	9–11	≥12
Water /flavored					
Original	-	100	-	-	^c^
Modified	95.0	-	5.0	-	-
Tea and coffee					
Original	-	100	-	-	^c^
Modified	100	-	-	-	-
Fruit juice					
Original	99.3	-	0.3	0.3	^c^
Modified	0.7	2.4	25.2	62.2	9.4
Fruit nectar					
Original	-	-	17.6	82.4	^c^
Modified	-	-	5.9	2.9	91.2
Fruit flavored drink					
Original	19.2	7.7	38.5	34.6	^c^
Modified	-	12.8	3.8	19.2	64.1
Art. sweetened					
Original	1.3	88.8	6.3	3.8	^c^
Modified	-	86.3	7.5	1.3	5.0
Sweetened drinks					
Original	0.4	5.8	34.2	59.6	^c^
Modified	–	3.8	9.2	23.3	63.8

a *Food composition data from the Open Food Facts food composition database.*

b *Adaptation from original FSA algorithm to the Nutri-Score algorithm, see Box 2.*

c *In original FSA algorithm only 4 categories (quartiles) for beverages*.

**Table 3:** Discriminating performance of Nutri-Score: distribution of breakfast cereal types and equivalent products (%) across quintiles of the FSA-score distribution^a^, ^b^ as described in Julia et al. (20) (Tables 3, 4, *n* = 380).

	**Quintiles of FSA-score**					
	**Q1 (< -2)**	**Q2 (-1;3)**	**Q3 (4;11)**	**Q4 (12;16)**	**Q5 (≥17)**	***n***
**For cereal types**
Crunchy muesli	11.1	9.1	46.5	27.3	6.1	99
Chocolate cereals	-	9.0	84.3	6.7	-	89
Light cereals	5.0	11.7	71.7	11.7	-	60
Filled cereals	-	-	37.5	45.0	17.5	40
Honey cereals	-	5.7	65.7	28.6	-	35
Cornflakes/plain	20.0	5.0	70.0	5.0	-	20
Muesli flakes	78.6	14.3	7.1	-	-	14
Oat flakes	66.7	16.7	16.7	-	-	12
Fiber-rich flakes	27.3	27.3	45.5	-	-	11
**For equivalent products**
Chocolate-flavor						
Chocolate wheat flakes	4.5	4.5	81.8	9.1	-	22
Chocolate puffed rice	-	7.7	76.9	15.4	-	13
Chocolate puffed cereal	-	15.0	85.0	-	-	20
Light cereals
Choc light cereals	-	15.4	69.2	15.4	-	13
Fruit light cereal	9.1	9.1	81.8	-	-	11
Unflavoured light cereals	-	11.1	88.9	-	-	9
Filled cereals
W/ milk chocolate	-	-	33.3	22.2	44.4	9
W/ chocolate hazelnut	-	-	31.3	68.8	-	16

a *Food composition data from brand sites, online supermarkets and consumer's nutritional websites.*

b *Cut-offs based on quintile distribution as described in Julia et al. (19)*.

Given the absence of a gold standard, the ability to discriminate nutritional quality was evaluated using a pragmatic approach: the discriminating performance was considered adequate if products were distributed over at least three classes of Nutri-Score (20). For discrimination between equivalent products of different brands, the criterion of “at least three classes” was later adapted to “at least two classes” (21).

The WHO specifies content validity as a classification of products, rather than classification of foods “as consumed” (12). Yet, Julia et al. (19) conducted both an unweighted and weighted analysis, i.e., weighting was done in such a way that the scores for products that were consumed in larger amounts were weighted more heavily. Although weighted and non-weighted results were fairly consistent for food groups such as “meat, fish & eggs” or “composite foods,” some discrepancies can be observed, for example, in “cereals, legumes & potatoes,” and “milk & dairy.”

Overall, Nutri-Score showed a high discriminating performance, as it was able to discriminate across and within PNNS food groups, but also across equivalent products from different brands, such as breakfast cereals (19–21). However, the studies did not take into account the diversity of products within different types of product groups and their distribution across the five classes. For example, the presence of at least three classes of an FOP label may be useful for breakfast cereals, but not for eggs. Also, if one class contains 90% of a type of breakfast cereals, and the surrounding classes contain only 5% each, the discriminating performance may still be considered limited.

Furthermore, the distributions of the PNNS food group across quintiles of the FSA-score distribution in the French NutriNet Santé food composition database and in the Open Food Facts database were quite consistent (Tables 1, 2). For example, in both studies, fruits and vegetables had the lowest FSA-score (indicating better nutritional quality, see Box 1), and 90–95% of products fell in the first and second quintile of the score distribution. In contrast, “sugary snacks” received the highest FSA-score and the majority of sugary products (79–86%) fell into the fourth and fifth quintile of the score distribution. Interestingly, both composite foods and nuts scored relatively low, indicating better nutritional quality despite their potentially high sodium levels, and in the beverages category, fruit juices scored consistently lower–thus healthier than water (19, 21). The classification between food categories was consistent across studies (see Supplementary material 3): milk & yogurt classified lower, and thus had a higher nutritional quality than dairy desserts and ice cream. Moreover, unprocessed meat and fish had a higher nutritional quality than processed meat (19, 21). However, the studies did not provide insight into the classification of different types of foods within a food category– e.g., wholegrain products vs. refined grain products, or whole milk vs. skimmed and semi-skimmed milk. The studies also did not give examples of actual foods in each class to allow a comparison of healthfulness on that level. In the development of the Choices criteria, for example, indicator foods were used to assess compliance with the criteria (9).

## Nutri-Score's convergent validity: Adherence to French dietary guidelines

Julia et al. (21) not only examined content validity but also took the validation process one step further, by examining convergent validity. Convergent validity refers to the consistency between different measures: how does the categorization of products using the Nutri-Score algorithm compare to the categorization of products using another system (12), in this case, the French dietary guidelines (31).

In their study, Julia et al. (21) noted discrepancies between categorization using the original FSA score and the French dietary guidelines for beverages, dried fruits, nuts, fats and cheese (see Box 2). Not all guidelines were reported on, for instance, no reference was made to the dietary guideline for wholegrain products.

Box 2 **Convergent validity of the FSA/Ofcom model and proposed adaptations to the Nutri-Score model.**

To assess convergent validity, Julia et al. (21) compared the French national dietary guidelines (31) with the 5-category classification using the FSA/Ofcom model. Discrepancies were noted in the following guidelines:

^*^ at least five fruits & vegetables a day–dried fruits as a component of this food group is considered a snack and not recommended

^*^ 3 servings of milk and dairy products per day–cheese is considered a good source of calcium and is included in recommendation

^*^ added fats: limit consumption; vegetable added fats: favor fats of vegetable origin–original FSA score does not allow for the differentiation in types of fats

^*^ salt: limit consumption–nuts are considered a salty snack and therefore not recommended

^*^ beverages: drink water as desired; limit sweetened beverages: no more than 1 glass per day–original FSA score does not reflect the recommendations and show low variability (only quartiles with original score)

The following guidelines were not reported:

^*^ bread, cereals, potatoes and legumes at each meal according to appetite

^*^ preferentially choose whole grains and wholegrain breads

^*^ meat and poultry, seafood and eggs: 1 to 2 per day

^*^ seafood at least twice a week

^*^ alcohol: ≤ 2 glasses for women, ≤ 3 glasses for men (not relevant, alcohol not included in FSA/Ofcom model)

^*^ sugary foods: limit consumption

Based on these discrepancies, the following adaptations were proposed:

Adaptations to points allocation

All foods & beverages: calculate the content of fruit, vegetable, legumes and nuts, excluding dried fruits and nuts

Fats & oils: adapt points for saturated fats:4 g/100 g ascending step

Beverages: adapt points for energy and sugar:

– energy: 30 kJ/100 g ascending step

– sugar: 1.5 g/100 g ascending step

Adaptations to score calculation

Dairy:

Total score = N-points – P-points

Categorization

Food categories similar to original categories; new categories for beverages^*^:

Foods:

1/A green ≤ 2

2/B yellow−1–3

3/C orange 4–11

4/D pink 12–16

5/E red ≥17

Beverages:

1/A green ≤ 0

2/B yellow 1–4

3/C orange 5–8

4/D pink 9–11

5/E red ≥12

^*^ Food categories were based on the distribution of FSA-scores (quintiles) in the NutriNet Santé food composition table (*n* = 3,508); the process to define the beverage categories was not reported, but categories were presumably based on the distribution of FSA-scores for beverages in the Open Food Facts food composition database (only for products marketed in France, *n* = 793).

The authors proposed a number of adaptations to the algorithm to address the observed discrepancies. Table 2 shows the shift in distribution for these food groups after the proposed adaptation of the algorithm. For instance, after the modification only a small proportion of fruit juices fell into the A and B categories (i.e., 3.1%) compared to the original score (i.e., 99.3%). The final adaptations to the algorithm before introducing the Nutri-Score label, as determined by the French High Council of Public Health (22), are described in Supplementary material 2.

## Nutri-Score's predictive validity in France: Prospective associations with disease risk

As a last and most complex step in the validation process, the WHO describes the predictive validity as follows (12): “In this most advanced type of testing, nutrient profiling criteria are applied to population dietary data, and these data are then used to compare health risks across population segments with better or worse diet quality, based on the nutrient profiling criteria.” To assess predictive validity for Nutri-Score, first a dietary index was developed and validated by examining the associations with nutrient intakes (see Box 3) (32, 33). Notably, this dietary index was based on the original FSA/Ofcom algorithm, whereas the subsequent studies examining the dietary index and disease risk, were based on modified algorithms, more specifically on the Nutri-Score algorithm #1 and #2 (see Figure 2). These Nutri-Score algorithms used the non-starch polysaccharides method for fiber content, which was adapted in 2018 to adhere to the French government's new regulations, setting the AOAC method as the reference method for fiber content (personal communication Dr. Julia) (34). However, the various adaptations do not seem to have affected the results to a large extent, as it was shown that associations with BMI, overweight and obesity were more or less comparable in four variants of the FSA dietary index, including the one using the Nutri-Score algorithm (29). For the purpose of this paper, we will refer to the Nutri-Score dietary index (NS-DI) if the index is based on one of the variations in the Nutri-Score algorithm, and to the FSA dietary index (FSA-DI) if the index is based on the original FSA/Ofcom model.

Box 3 **Computation and validation of the FSA-dietary index**

The **FSA-dietary index (FSA-DI)** is an aggregated FSA-score at the individual level and is calculated as follows:

1) For each food and beverage the individual consumes, the FSA-score is computed

2) The FSA-score of each food and beverage consumed is multiplied by the energy intake from that food or beverage

3) All FSA-scores are subsequently added up and the resulting summary score is divided by the total amount of energy consumed:

FSA-DI = Σ(FSA-score_i_ * energy intake_i_) / Σ(energy intake_i_)

The FSA-DI was **validated** in two populations: participants of the NutriNet Santé study (33) and participants of the SUVIMAX study (34). FSA-DI (in quartiles) was validated against various nutritional indicators and the adherence to the French dietary guidelines. As to be expected, significant associations were observed with macronutrients that are part of the algorithm, as well as with fiber and sodium that are also part of the algorithm. A negative association was found only with sugar (higher sugar intakes at lower FSA-DI). The authors suggested that this may be explained by the fact that simple sugars are present in basic foods such as milk, fruits and vegetables. In terms of added sugars, the association was as expected. Significant associations were also observed with micronutrients. Reported intakes were adjusted for energy intake, age and sex, which bolster the observations given that it controls for the variation introduced by sex and age (different consumption patterns for males and females and for older and younger people) and by energy intake which is correlated to nutrient intake.

A lower FSA-DI, indicating better diet quality, was positively associated with the Programme National Nutrition Santé guideline score (PPNS-GS), a score reflecting adherence to French dietary guidelines. However, there was no association between the FSA-DI and adherence to the specific recommendations for dairy products, meat, poultry, seafood and eggs, as well as for added vegetable fats. Interestingly, only 19% of the group with the lowest FSA-DI adhered to the wholegrain recommendation.

Until 2021, a total of 7 studies investigated the prospective association between the NS-DI and disease risk in two different study populations in France (Table 4). Outcome variables included BMI (including overweight/obesity risk) (24, 29), metabolic syndrome risk (23), cardiovascular disease (CVD) risk (26, 27), and cancer risk (25, 28). Overall, the results of the predictive validity studies suggest a significant, albeit small association between the highest dietary index (reflecting lower nutritional quality of the diet) and disease risk, particularly with regard to overall CVD (26, 27) and total cancer risk (25) (Table 4). As the studies were performed in a relatively healthy population, associations may have been underestimated. As volunteers in a nutrition and health-related study, participants of the SUVIMAX and NutriNet Santé study were likely to have more health-conscious behaviors, including better food choices. These are limitations that are shared by many prospective cohort studies, and could have weakened associations, but this ultimately depends on the distribution of the Nutri-Score dietary index in the general population. Strengths of all studies include their large sample sizes and the use of repeated 24-h recalls, which can be considered a relatively accurate measure for dietary intake. Also, all four SUVIMAX studies included data from a long-term follow-up of at least 13 years.

**Table 4:** Results of predictive validity studies for the Nutri-Score algorithm in the French context: multivariable associations of the Nutri-Score dietary index with overweight, obesity and metabolic syndrome [odds ratios (OR) with 95% confidence intervals] and with cardiovascular disease risk, cancer risk and mortality [hazard ratios (HR) with 95% confidence intervals].

**Reference**	**Study population**	**Version of the Nutri-Score (NS) algorithm**	**Categorization of the dietary index**	**Outcome variables**	**Results: multivariable adjusted, significant associations**
Julia et al. (23)	SUVIMAX; *n* = 3,741	NS model #1	Sex-specific quartiles and continuous	Metabolic syndrome (MetS) and metabolic syndrome traits: waist circumference, triglycerides, high density lipoprotein (HDL), diastolic blood pressure (DBP), systolic blood pressure (SBP), fasting glucose	MetS: OR_Q4vs.Q1_ = 1.43 (1.08; 1.89), P_trend_ 0.02 MetS traits: *DBP per quartile :* Q1 77.2 (76.4; 77.9) Q2 77.8 (77.0; 78.6) Q3 77.7 (77.0; 78.5) Q4 78.7 (77.9; 79.5); P_trend_ 0.01 *SBP per quartile:* Q1 124.9 (123.7; 126.1) Q2 125.8 (124.6;126.9) Q3 125.8 (124.6;127.0) Q4 127.1 (125.9;128.3); P_trend_ 0.01
Donnenfeld et al. (25)	SUVIMAX; *n* = 6,435	NS model #1	Sex-specific quintiles and continuous	Cancer overall Prostate cancer Breast cancer	Cancer overall: HR_Q5vsQ1_ = 1.34 (1.00; 1.81), P_trend_ 0.03 HR_1−pointincrement_ = 1.08 (1.01; 1.15), P 0.02
Julia et al. (24)	SUVIMAX; *n* = 4,344	NS model #1	Sex-specific quartiles and continuous	BMI change, overweight, obesity	BMI change: ΔBMI (kg/m^2^) _Q4vsQ1_ = 0.70 (0.01; 1.38), P_linearcontrasts_ 0.04 Overweight: Men: OR_Q4vs.Q1_ = 1.61 (1.06; 2.43), P_linearcontrasts_ 0.02 OR_1−pointincrement_ = 1.13 (1.02; 1.25), P 0.02 Women: OR_Q3vs.Q1_ = 0.70 (0.51; 0.96) OR_Q4vs.Q1_ = 0.74 (0.54; 1.02), P_linearcontrasts_ 0.04 Obesity: Men: OR_Q4vs.Q1_ = 1.91 (1.12; 3.26), P_linearcontrasts_ 0.01 OR_1−pointincrement_ = 1.16 (1.02; 1.31), P 0.02 Women: OR_Q3vs.Q1_ = 0.54 (0.32; 0.91)
Adriouch et al. (26)	SUVIMAX; *n* = 6,515	NS model #1	sex-specific quartiles & continuous	CVD	CVD: HR_Q4vsQ1_ = 1.61 (1.05; 2.47), P_trend_ 0.03 HR_1−pointincrement_ = 1.14 (1.03; 1.27), P 0.01
Adriouch et al. (27)	NutriNet-Santé; *n* = 76,647	NS model #2	Sex-specific quartiles and continuous	CVD, coronary heart disease, stroke	CVD: HR_Q4vsQ1_ = 1.40 (1.06; 1.84), P_trend_ 0.01 HR_1−pointincrement_ = 1.08 (1.03; 1.13), P 0.001 Coronary heart disease: HR_Q4vsQ1_ = 1.62 (1.12; 2.35), P_trend_ 0.01 HR_1−pointincrement_ = 1.09 (1.03; 1.16), P 0.005
Deschasaux et al. (28)	NutriNet-Santé; *n* = 46,864	NS model #2	Quintiles and continuous	Breast cancer	Breast cancer overall: HR_Q2vsQ1_ = 1.43 (1.08; 1.90) HR_Q3vsQ1_ = 1.43 (1.07; 1.91) HR_Q4vsQ1_ = 1.79 (1.35; 2.38) HR_Q5vsQ1_ = 1.52 (1.11; 2.08), P_trend_ 0.002 HR_1−pointincrement_ = 1.06 (1.02; 1.11), P 0.005 Premenopausal women: HR_Q4vsQ1_ = 2.76 (1.45; 5.26) HR_Q5vsQ1_ = 2.46 (1.27; 4.75), P_trend_ 0.004 HR_1−pointincrement_ = 1.09 (1.01; 1.18), P 0.03 Postmenopausal women: HR_Q4vsQ1_ = 1.57 (1.13; 2.18) HR_Q5vsQ1_ = 1.25 (0.85; 1.84), P_trend_ 0.09 HR_1−pointincrement_ = 1.05 (1.00; 1.11), P 0.06
Egnell et al. (29)	NutriNet-Santé; *n* = 71,403	NS model #2	Sex-specific tertiles and continuous	BMI change, overweight, obesity	ΔBMI not reported Overweight: HR_Q2vsQ1_ = 1.13 (1.05; 1.22) HR_Q3vsQ1_ = 1.27 (1.17; 1.37), P_trend_ <0.0001 HR_1−pointincrement_ = 1.02 (1.01; 1.03), *P* < 0.0001 Obesity: HR_1−pointincrement_ = 1.03 (1.01; 1.06), P 0.004

Yet, from a methodological point of view it may be questioned whether the studies reporting on predictive validity are able to predict Nutri-Score's actual association with disease or health over time, as they are based on consumption data that was not driven by Nutri-Score. Furthermore, the methodological approach differed across all predictive validity studies: some studies used sex-specific quartiles (23, 24, 26, 27), while other studies used sex-specific quintiles (25, 28), and one study even used sex-specific tertiles (29).

Trend analyses were done using a continuous value for the dietary index as well as using either the median (23), mean (24), or ordinal values (25–28) of the quartiles/quintiles, which makes comparison between studies problematic. Generally, using the mean or median values is preferred over using the ordinal values because it better reflects the data. At the same time, using ordinal values, as well as fitting a continuous linear trend to data that in fact may be rather curved can be considered a conservative estimation, as the residual variance will increase. In the study by Egnell et al. (29), neither the methodology of the trend analysis, nor the actual change in BMI was reported. Moreover, in all but one study (25), statistical analyses were adjusted for energy intake. One could debate whether adjustment for energy intake is required here. In their study, Drewnoski et al. (35) observed a high correlation between the FSA-score of the original WXYfm model and the energy density of foods, suggesting that the score provides rather more information on calories than on nutrient composition.

## Validation in the European context

Over the years, the validation process of Nutri-Score has been extended to the European level, with a number of studies examining either content validity (36–39) (Table 5) or predictive validity (40–42) (Table 6). The content validity studies [two of which remain unpublished, but are presented online (38, 39)], generally showed adequate discriminating performance within food groups–at least three Nutri-Score classes represented–but not necessarily within food categories. For example, less than three Nutri-Score classes were occasionally observed for dairy desserts in the milk & dairy group, for pastries or chocolate products in the sugary snacks group and for sandwiches or soups in the composite dishes group (36–39). Also, as previously mentioned, with no indicator foods, the actual healthfulness of foods in the different Nutri-Score classes remains unknown.

**Table 5:** Country-specific distributions (%)^a^, ^b^ of food groups across Nutri-Score classes as reported for Germany in Szabo et al. (36) and for Spain, Switzerland, Belgium, Italy, UK, The Netherlands, Sweden, Austria, Finland, France, Poland and Portugal in Szabo et al. (39)^*^.

		**Nutri-Score nutritional quality category**					
**Food group**		**A**	**B**	**C**	**D**	**E**	***n***
**Fruits and vegetables**							
	Germany	61.4	18.4	18.0	1.9	0.4	527
	Spain	63.9	12.6	20.4	2.9	0.3	4,244
	Switzerland	60.9	15.3	22.2	1.5	0.1	946
	Belgium	59.4	15.6	23.3	1.6	0.2	945
	Italy	67.3	19.0	12.0	1.4	0.4	284
	UK	66.7	14.4	16.2	2.7	0	487
	Netherlands	70.2	13.7	14.5	1.5	0	131
	Sweden	48.7	14.1	34.6	2.6	0	78
	Austria	61.0	18.1	17.1	3.8	0	105
	Finland	65.0	7.5	27.5	0	0	40
	France	59.7	14.6	21.8	3.6	0.4	17,253
	Poland	55.6	27.3	14.1	3.0	0	99
	Portugal	59.3	15.3	20.3	5.1	0	59
**Cereals, legumes and potatoes**							
	Germany	49.4	19.9	18.9	10.5	1.4	1,396
	Spain	31.5	22.5	21.2	21.6	3.3	6,811
	Switzerland	44.1	18.5	21.7	13.5	2.2	2,274
	Belgium	40.3	18.3	23.8	14.7	2.9	1,795
	Italy	50.4	13.3	18.7	16.3	1.3	1,249
	UK	43.2	19.5	20.1	14.5	2.8	1,117
	Netherlands	51.4	15.3	19.5	13.3	0.4	451
	Sweden	50.2	20.5	13.9	12.5	2.9	273
	Austria	47.6	16.4	21	14.4	0.6	353
	Finland	60.0	19.5	15.0	4.5	1.0	200
	France	40.7	18.5	20.3	17.1	3.4	24,346
	Poland	58.2	11.9	22.2	6.9	0.8	261
	Portugal	37.1	16.9	27.3	16.5	2.2	267
**Milk and dairy**							
	Germany	12.9	18.1	23.5	42.4	3.2	1,875
	Spain	11.3	26.7	17.4	39.4	5.2	7,868
	Switzerland	10.9	22.0	25.1	39.5	2.5	2,380
	Belgium	10.3	23.2	18.5	43.1	4.9	2,122
	Italy	15.9	30.4	24.1	26.5	3.2	1,205
	UK	15.2	22.3	21.5	37.2	3.8	1,056
	Netherlands	23.5	31.5	13.1	28.3	3.7	375
	Sweden	21.8	16.9	13.4	43.1	4.8	455
	Austria	9.3	28.7	19.4	38.3	4.3	397
	Finland	21.4	22.9	13.4	38.8	3.5	201
	France	7.3	17.9	23.5	46.5	4.8	33,416
	Poland	10.8	38.7	14.4	29.0	7.1	507
	Portugal	21.8	35.6	16.3	24.6	1.7	289
**Meat, fish and eggs**							
	Germany	7.7	14.1	13.4	37.6	27.2	688
	Spain	9.6	14.3	22.1	36.6	17.3	6,716
	Switzerland	12.4	15.5	19.0	39.9	13.2	1,213
	Belgium	11.4	14.7	24.7	33.7	15.5	1,464
	Italy	8.6	17.2	20.8	43.0	10.5	419
	UK	20.7	23.3	18.0	27.2	10.9	707
	Netherlands	8.5	15.1	17.9	34.9	23.6	106
	Sweden	17.2	7.0	15.9	36.9	22.9	157
	Austria	7.9	12.5	17.8	34.2	27.6	152
	Finland	14.3	19.6	24.1	27.7	14.3	112
	France	13.1	13.5	20.3	32.6	20.5	35,721
	Poland	5.8	9.7	24.5	41.3	18.7	155
	Portugal	7.0	21.1	29.6	38.0	4.2	71
**Sugary snacks**							
	Germany	0.7	2.3	3.6	22.1	71.3	1,745
	Spain	2.4	5.3	12.7	37.2	42.5	9,555
	Switzerland	1.2	3.9	10.0	32.2	52.6	3,262
	Belgium	1.7	4.0	11.5	31.5	51.3	2,686
	Italy	1.9	2.9	19.3	39.1	36.8	1,472
	UK	1.1	2.8	8.6	38.6	48.9	1,539
	Netherlands	1.4	3.4	13.7	33.6	48.0	563
	Sweden	2.7	2.9	8.8	28.7	56.9	376
	Austria	1.7	3.3	7.8	23.8	63.4	424
	Finland	0.3	2.9	4.8	32.7	59.4	315
	France	0.8	2.7	11.6	39.1	45.8	52,951
	Poland	1.2	2.8	15.9	24.2	56.0	327
	Portugal	2.8	5.2	11.4	39.4	41.2	325
**Salty snacks**							
	Germany	1.5	1.9	19.4	63.4	13.8	413
	Spain	3.4	5.9	27.9	53.0	9.8	3,154
	Switzerland	8.6	7.5	35.3	38.9	9.7	745
	Belgium	3.8	6.7	34.1	45.6	9.9	766
	Italy	13.5	3.9	40.0	38.7	3.9	155
	UK	7.9	10.9	32.5	40.9	7.9	496
	Netherlands	12.9	9.3	39.3	34.3	4.3	140
	Sweden	5.3	3.5	13.2	71.9	6.1	114
	Austria	8.8	9.9	44.0	34.1	3.3	91
	Finland	18.2	0	31.8	40.9	9.1	22
	France	3.7	7.2	27.7	39.5	21.8	17,246
	Poland	2.3	3.8	33.1	58.5	2.3	130
	Portugal	4.2	4.2	38.0	47.9	5.6	71
**Fat and sauces**							
	Germany	2.1	2.7	26.7	48.8	19.7	619
	Spain	5.3	5.9	53.1	24.9	10.7	3,909
	Switzerland	6.4	7.4	33.1	38.6	14.5	1,186
	Belgium	3.7	3.8	27.8	42.8	22.0	1,223
	Italy	6.6	5.7	31.5	27.8	28.4	454
	UK	4.1	8.1	38.3	37.9	11.6	689
	Netherlands	6.2	5.3	27.9	45.6	15.0	226
	Sweden	3.7	5.3	25.9	44.4	20.6	189
	Austria	9.1	9.1	34.7	36.4	10.8	176
	Finland	10.1	1.4	18.8	49.3	20.3	69
	France	4.5	6.0	32.0	38.5	18.9	18,460
	Poland	2.5	1.4	19.3	53.9	22.9	280
	Portugal	3.2	7.4	20.0	49.5	20.0	95
**Composite foods**							
	Germany	8.6	21.5	48.0	20.8	1.1	452
	Spain	9.6	19.6	35.8	31.2	3.8	2,350
	Switzerland	13.7	25.1	38.6	20.1	2.5	1,067
	Belgium	13.0	31.8	36.6	16.9	1.6	999
	Italy	10.6	17.4	33.2	34.7	4.1	340
	UK	28.7	35.3	21.2	12.5	2.3	655
	Netherlands	12.3	16.1	47.1	20.0	4.5	155
	Sweden	10.8	25.9	48.5	15.0	0	293
	Austria	7.8	18.4	50.8	19.6	3.4	179
	Finland	12.8	22.9	37.6	26.6	0	109
	France	16.1	30.3	31.0	19.1	3.4	24,106
	Poland	3.1	28.1	50.0	14.6	4.2	96
	Portugal	24.4	24.4	31.1	17.8	2.2	45
**Beverages**							
	Germany	28.1	7.2	19.8	12.7	32.1	872
	Spain	32.3	13.3	22.6	15.0	16.7	2,402
	Switzerland	11.1	9.6	21.5	20.1	37.6	1,268
	Belgium	16.9	11.0	23.4	21.1	27.6	1,241
	Italy	25.4	7.5	19.8	9.3	38.0	389
	UK	13.2	15.3	33.7	16.7	21.1	478
	Netherlands	17.9	10.1	25.7	21.2	25.1	179
	Sweden	11.0	12.3	13.5	12.3	51.0	155
	Austria	13.0	7.1	22.1	22.7	35.1	154
	Finland	16.7	12.5	34.7	15.3	20.8	72
	France	8.7	8.8	24.2	16.7	41.7	16,237
	Poland	22.1	7.7	15.4	15.9	38.9	208
	Portugal	29.1	7.3	14.6	23.2	25.8	151

a *Food composition data from the Open Food Facts food composition database.*

b *Percentages reported were rounded to the nearest decimal for the present review.*

* *Data was published on the same website and as an update to Szabo et al. (38), and encountered after the screening phase of the current study. It was included in this review instead of Szabo et al. (38) as it contained more recent and a larger quantity of product data, including data from five additional countries*.

**Table 6:** Results of predictive validity studies for the Nutri-Score algorithm in the EPIC (40, 41) and SUN (42) studies: multivariable associations of the Nutri-Score dietary index with cancer risk and mortality [hazard ratios (HR) with 95% confidence intervals].

**Reference**	**Study population**	**Version of the Nutri-Score (NS) algorithm**	**Categorization of the dietary index**	**Outcome variables**	**Results: multivariable adjusted, significant associations**
Deschasaux et al. (40)	EPIC cohort; *n* = 471,495	NS model #2	Sex-specific quintiles and continuous per 2-point increment	Total cancer Colorectal cancer Bladder cancer Kidney cancer Upper aerodigestive tract cancer Lung cancer Stomach cancer Pancreas cancer Liver cancer Prostate cancer Breast cancer Endometrial cancer Cervical cancer Ovary cancer	Total cancer: HR_Q4vs.Q1_ = 1.06 (1.03; 1.09) HR_Q5vs.Q1_ = 1.07 (1.03; 1.10), P_trend_ <0.001 HR_2−pointincrement_ = 1.02 (1.01 ; 1.03), *P* < 0.001 Colorectal cancer: HR_Q4vs.Q1_ = 1.12 (1.02; 1.22) HR_Q5vs.Q1_ = 1.11 (1.01; 1.22), P_trend_ 0.02 HR_2−pointincrement_ = 1.03 (1.00 ; 1.06), P 0.03 Upper aerodigestive tract: HR_2−pointincrement_ = 1.07 (1.01 ; 1.14), P 0.03 Stomach cancer: HR_2−pointincrement_ = 1.10 (1.02 ; 1.18), P 0.01 Prostate cancer: HR_2−pointincrement_ = 1.03 (1.00 ; 1.06), P 0.04
Deschasaux et al. (41)	EPIC cohort; *n* = 501,594	NS model #2	Sex-specific quintiles and continuous per 1-standard deviation (SD) increment	All-cause mortality Cause-specific mortality: Non-external External Cancer Circulatory diseases Respiratory diseases Digestive diseases	All-cause mortality: HR_Q5vsQ1_ = 1.06 (1.03; 1.09), P_trend_ <0.001 HR_1−SDincrement_ = 1.02 (1.01; 1.03), *P* < 0.001 Non-external mortality: HR_Q5vsQ1_ = 1.07 (1.03; 1.10), P_trend_ <0.001 HR_1−SDincrement_ = 1.03 (1.02; 1.04), *P* < 0.001 Cancer mortality: HR_Q5vsQ1_ = 1.08 (1.03; 1.13), P_trend_ <0.001 HR_1−SDincrement_ = 1.03 (1.01; 1.04), *P* < 0.001 Circulatory disease mortality: HR_1−SDincrement_ = 1.02 (1.00; 1.04), P 0.03 Respiratory disease mortality: HR_Q2vsQ1_ = 1.15 (1.01; 1.31) HR_Q3vsQ1_ = 1.16 (1.01; 1.32) HR_Q4vsQ1_ = 1.27 (1.11; 1.45)
					HR_Q5vsQ1_ = 1.39 (1.22; 1.59), P_trend_ <0.001 HR_1−SDincrement_ = 1.11 (1.06; 1.15), *P* < 0.001 Digestive disease mortality: HR_Q5vsQ1_ = 1.22 (1.02; 1.45), P_trend_ 0.03 HR_1−SDincrement_ = 1.08 (1.02; 1.14), P 0.01
Gómez-Donoso et al. (42)	SUN cohort; *n* = 20,503	NS model #4	Sex-specific quartiles and continuous per 2-point increment	All-cause mortality Cancer mortality Cardiovascular mortality	All-cause mortality: HR_Q2vsQ1_ = 1.37 (1.03; 1.83) HR_Q3vsQ1_ = 1.43 (1.06; 1.94) HR_Q4vsQ1_ = 1.82 (1.34; 2.47), P_trend_ <0.001 HR_2−pointincrement_ = 1.19 (1.08; 1.32) Cancer mortality: HR_Q2vsQ1_ = 2.08 (1.37; 3.15) HR_Q3vsQ1_ = 1.99 (1.30; 3.06) HR_Q4vsQ1_ = 2.44 (1.54; 3.85), P_trend_ <0.001 HR_2−pointincrement_ = 1.24 (1.09; 1.41)

Table 5 provides an overview of the distribution of Nutri-Score classes within food groups for Germany, Spain, Switzerland, Belgium, Italy, UK, Netherlands, Sweden, Austria, Finland, France, Poland and Portugal, based on the Open Food Facts database (36, 39). The study of Dréano-Trécant et al. (37) used a different food database (the EUROFIR database) that did not allow for a similar food group classification. The main results of this study are described in Supplementary material 4. While the studies based on the Open Food Facts database made sure to only include countries with more than 1,000 products available, the number of foods included in the analyses generally varied significantly across countries and food groups and categories, which may be the result of not using an official, validated country-specific database. In the Open Food Facts database, data is voluntarily entered by anyone who wishes to do so, and is derived from stores, including national brands, store brands and discount brands (43). In the EUROFIR database, data is retrieved from various sources including research institutes, food quality organizations and commercial organizations (37). In both databases, representativeness for the actual supermarket food supply are unknown (36, 37).

In the country-specific analyses, the majority of fruit and vegetables were classified favorably as Nutri-Score A or B (64 to 86%), while the majority of sugary snacks was classified D or E (77 to 92%) (Table 5) (36, 38, 39). Thus, these analyses generally showed high content validity, with recommended food groups scoring favorably and non-recommended food groups scoring more unfavorably. However, this was claimed as evidence for convergent validity, while no extensive analysis was conducted on the Nutri-Score's consistency with nutritional recommendations in the different countries. For example, German guidelines recommend wholegrain choices for cereals, daily consumption of milk and dairy, and the use of vegetable oils instead of animal fats (44), but these recommendations were not evaluated in detail. Moreover, a Dutch evaluation study commissioned by the Dutch Ministry of Health, showed a number of discrepancies between the Nutri-score categorization and the Dutch dietary guidelines, in particular, pertaining to the recommendations for bread, vegetables, cheese and fats (45). Another Dutch study (*n* = 2,299 products) found discrepancies for cheese, ready meals, soups and sauces (46). This study for example showed that ready meals with a green Nutri-Score A or B contained 2.9 g salt per portion on average (48% of the acceptable daily intake), and are thus not considered healthy according to Dutch dietary guidelines. Previous work suggests that nutrition labels showing a green color enhance perceived healthfulness of products and could even lead to overconsumption (47). Thus, consumers may be misled into thinking that ready meals are healthy, while they are generally high in saturated fat, energy and salt, and consuming them may negatively impact consumers' overall health and lead to overweight (48).

For the predictive validity studies in European context, results were similar to the previous studies in French populations (40–42). Overall, a higher dietary index, representative of lower nutritional dietary quality, was associated with a higher risk of cancer (40) and mortality (41) in the multinational EPIC cohort, and with higher risk of mortality in the SUN cohort (42) (Table 6). This was based on analyses of hazard ratios associated with higher vs. lower quartiles/quintiles of the dietary index or the continuous index score. The continuous analyses in hazard ratios were reported per 2-point increment (40, 42) or per 1 standard deviation increment (41), instead of per 1-point increment as in previous French studies. The SUN cohort may be less representative for a general population, being a relatively young cohort (38 ± 12 years upon inclusion) of university graduates, with normal BMI, that showed no variation across the dietary index quartiles (42). The EPIC cohort as a multi-country study showed more variation in educational level and BMI (40, 41). Here, BMI was inversely associated with a higher dietary index, which may suggest reversed causality–and thus an underestimation of true association, as people with higher risk of disease may have adapted their dietary intake. Interestingly, in sensitivity analyses there appeared to be no associations between the Nutri-Score dietary index and mortality in overweight and obese individuals (41). A similar observation was done in the study of Adriouch et al. (26), examining risk of cardiovascular diseases. These results may suggest a limited influence of dietary adaptation based on the Nutri-Score nutrient profile model in overweight and obese individuals.

## Reflection

Nutri-Score's development and validation process may be considered extensive compared to the other major FOP labels currently in use in Europe, i.e., Keyhole, Choices and MTL. The Keyhole logo was introduced without validation in 1989, but nowadays its criteria are evaluated every 5 to 6 years by a scientific committee with members from all participating countries (49). The Choices criteria were developed in 2006 and have been regularly updated by an international scientific committee (50) since. Evaluations include validations using indicator foods, modeling studies to estimate improvements in habitual nutrient intakes, and the validation of a recent extension of the Choices criteria into a five-level system that can serve other policy purposes such as reformulation (6, 51). The MTL logo was recently updated (7), after being formally introduced in 2013 following years of research and stakeholder consultation, with criteria based on health claim regulations (7, 52).

Despite years of research on nutrient profiling, validation of a nutrient profile model, in any form, remains a difficult issue, as no consensus has yet been reached on a gold standard. This holds especially for convergent validity, but also for content validity–how can one determine whether a classification is indeed based on healthfulness? In our opinion, an indicator foods approach (53) allows for better insight in classification, as not only the scoring or calculation method of a nutrient profile model determines the scheme's ability to rank foods according to healthfulness, but also the reference quantity, choice and balance of nutrients included, and categories of food taken into account. Using serving size instead of a “per 100 g or 100 ml” reference basis would better reflect on the quantity of food typically consumed, which is an essential determinant of the potential of a product to adversely affect overall dietary balance (54). The “per 100 g or 100 ml” reference unit of the Nutri-Score may lead to false projections of healthfulness. For instance, as described earlier, ready meals may get relatively favorable scores while they are high in salt and saturated fat, because they are scored based on nutrient levels per 100 g. However, they are consumed in portion sizes larger than 100 g, and thus their final score may be largely underestimated (46). Most currently existing FOP labels are not based on serving size, as there is a lack of standardization and regulation of serving sizes for different food groups at EU level. Since serving sizes are much more meaningful to consumers, further exploration of their harmonization at EU level is warranted. Presenting levels of critical nutrients per serving of a product on the front of the pack (e.g., as applied by the MTL) may also help consumers get more insight into the nutritional quality per serving consumed. Besides this, the balance of positive vs. negative nutrients is another concern, as addition of positive components such as fiber or protein may improve the Nutri-Score of a product without changing its unfavorable composition (54). Nutrient profile models should ideally include only a limited set of nutrients to avoid complexity and difficulty of adaptation (54). Yet, one could argue whether Nutri-Score actually represents the healthfulness of foods, as several nutrients or dietary components that may be either favorable or unfavorable for health are not evaluated by the label (54). Including certain components such as vitamins or minerals may serve an additional goal to public health importance, i.e., help discriminate better between food products within a food group, or serve as markers for specific food groups (55). Using a category-based nutrient profile model would enable the inclusion of specific sets of nutrients for food groups in the overall diet and make adaptions based on country-specific dietary recommendations easier (54). Moreover, such models allow for better comparability of portion size, frequency of intake and pattern of consumption of products within a food group. Arguably, Nutri-Score includes adjusted score calculations for only a relatively small number of food groups, i.e., added fats, cheeses and beverages. This may make adaptation more complex as changing one component in the scheme may affect the scoring for several food groups (54).

A recurring problem with convergent validity–usually assessed by comparing the classification with one construct against a classification with another construct, be it expert-based or based on dietary guidelines–is the circularity in the argument. Such circularity exists by definition if similar criteria or nutritional recommendations are used in both constructs (56). Perhaps a better term, at least for convergent validity, would be “calibrated” rather than “validated.” Research on the Nutri-Score's calibration in the European context is still in its infancy, and future work analyzing the Nutri-Score's agreement with dietary guidelines of other countries is warranted. Interestingly, the calibration of the Australia's and New Zealand's Health Star Rating (HSR), which is based on the same FSA/Ofcom algorithm underpinning Nutri-Score, showed similar issues as the French analyses of the Nutri-Score with regard to fat and dairy products. For the HSR, adjustments to the computation and cut-offs were also required to ensure alignment with the national dietary guidelines (57). In its 5-year review process, the HSR technical advisory group established the misalignment between the HSR and the Australian dietary guidelines to be 13 to 26%, depending on the dataset used and the applied cut-off (58). Observed algorithmic “failures” included products with high levels of sodium receiving a high star rating and thus suggesting high nutritional quality despite their high sodium levels (59), an issue also observed for Nutri-Score (46). Other reported issues were the high ratings received by fruit juices and breakfast cereals containing more than 25 grams of sugar per 100 grams (60), but also the rewarding of foods only for dietary fiber, not wholegrain, leading to inadequate differentiation between wholegrain and refined grain foods (61).

While studies supporting the health effects of the HSR are lacking (62), the predictive validity studies for the Nutri-Score algorithm do suggest an association with cancer (25, 41), cardiovascular disease (26, 27) and mortality (42, 43). A recent study in the Norfolk (UK) population of the EPIC cohort, using the original FSA/Ofcom model, found a small association between the consumption of less healthy foods and all mortality causes, but no association with cardiovascular disease or cardiovascular mortality (63). It is possible that the Nutri-Score algorithm was adapted in such a way that it better discriminates foods with respect to their association with CVD than the original FSA/Ofcom. However, as noted earlier, the predictive validation studies reported in this review may not provide a conclusive decision on Nutri-Score's actual impact on health. Ideally, one should assess consumption behavior driven by Nutri-Score, not by an analysis of consumers' diets quality using an “a posteriori” determined Nutri-Score index, and its association with disease over time. Moreover, the variability in methodological approaches–different populations, food databases and definitions or measures of the independent variable–requires additional research in different, more diverse populations, using consistent predictive analyses approaches. So far, only one study did compare various models originating from the FSA/Ofcom model (29). In this study, similar associations with weight gain, overweight and obesity were observed for all models, and these were slightly stronger for the Nutri-Score model.

While the current study reports outcomes of studies focusing on content, convergent and predictive validity specifically, it is important to discern between this type of validation–or calibration–of the model (the algorithm) and the validation–or evaluation–of its actual application (the label) (56). Crucially, the validity studies reported in this review alone do not provide a complete insight into the effectiveness of the Nutri-Score label, as they do not show whether Nutri-Score influences consumer purchasing behavior. The relevance of studying this additional step was previously noted by Julia et al. (21): “[…] the final effect of a FOP nutrition label based on the FSA-NPS [algorithm] would also depend on endorsement of the scheme by retailers and manufacturers and the actual format of the FOP label, which would also affect the perception, understanding and use of it by consumers.” Previous work has shown that Nutri-Score only had little impact on the nutritional quality of purchases in-store (64). The predictive validity studies included in this review made use of the dietary index, which is calculated from the habitual diet at one point in time, and not from a diet in which choices were made based on a FOP label. It is essential that future research is conducted on the actual effects of Nutri-Score based on adaptations of actual dietary intake in the target population as a result of buying products with the Nutri-Score or a label-induced change in the food supply. To date, the majority of studies into the effectiveness of the Nutri-Score label involve online surveys, asking consumers to classify primarily discretionary products–pizzas, cookies and breakfast cereals–based on their healthiness (65, 66). Consumer understanding of other product groups is lacking. In these surveys, different FOP labels were also compared with respect to consumer perception. A study on five different FOP labels indicated that the Nutri-Score label stood out the most, but it was less trusted and perceived to be more difficult to understand and not providing sufficient information (67). Overall, it is highly relevant to investigate how the Nutri-Score label will be used in an actual supermarket, and whether it will reach and help the intended target group choose healthier options. The drawback of many studies on FOP labels to date is that they are conducted in laboratory or experimental settings (68). These studies do not necessarily provide evidence of actual effectiveness of the Nutri-Score label, as participants in such studies are in a more “conscious mode,” i.e., they would make more deliberate choices than they would if they were not participating in a study (69). In fact, one recent real-life grocery shopping study that assessed the impact of different FOP labels on the nutritional quality of purchases, showed that the effect sizes were ~17 times smaller than those found in similar experimental studies (64). Ideally, more future work should look into methods to effectively measure the long-term impact of FOP labels, including Nutri-Score, in more naturalistic settings.

## Implications for European implementation of Nutri-Score

Current EU legislation allows voluntary FOP labeling as a visual representation of the mandatory back-of-pack nutrition declaration. However, discussions in the EU for establishing a mandatory FOP label are ongoing (2, 3). Currently, seven countries–France, Belgium, Luxembourg, Spain, Germany, Switzerland and the Netherlands–have implemented the Nutri-Score label or intend to implement it (10). While the WHO recommends adapting FOP labeling to national dietary guidelines (12) such adaptation was done only for France and not for the other countries so far. For instance, research from the Netherlands showed that the Nutri-Score is in line with the Dutch dietary guidelines recommending increased consumption of fruit and vegetable, pulses, and unsalted nuts (70), but there are also discrepancies (45, 46). Outside Europe, discrepancies with dietary guidelines were also reported on a FOP label based on the same algorithm (58–61). Studies on the Australian Health Star Rating, which uses a similar algorithm based on the FSA/Ofcom nutrient profile model, observed issues with the sodium criterium, allowing healthier scores (≥3.5 stars) for products with high levels of sodium (59), and wholegrain products not being adequately captured with the current dietary fiber criterium (61). This may have implications for population health, since high sodium and low intake of wholegrain foods are considered the two leading risks for mortality and disability-adjusted life years in the Lancet Global Burden of Disease Study (71).

The value of validating a nutrient profile model is not in the amount of studies, it is in their relevance. European implementation of Nutri-Score could benefit from content and convergent validity at the national level of the countries included, based on food databases that closely reflect the actual supermarket food supply in these countries. It is critical to evaluate to what extent the nutrient-based algorithm is aligned with the dietary guidelines in those countries, and what adaptations are required in the nutrient profile model to ensure alignment. One difficulty here is the across-the-board nutrient criteria the algorithm uses, criteria that may prove difficult to align with the food-based dietary guidelines that most countries have, as demonstrated for example by the discrepancies involving wholegrain products despite the fiber criterion in the algorithm. Additionally, it is essential to investigate what adaptations are required to help product innovation and reformulation, as this may be an even more important avenue to help consumers eat healthier diets (4). To date, it is unknown whether adapting the across-the-board criteria of Nutri-Score will support product reformulation for different food groups or whether food group-specific criteria are required. A Dutch analysis investigated to what extent different product improvement scenarios can initiate a shift in Nutri-Score and hence can be an incentive for reformulation (70). It was found that a reduction in sodium, saturated fat or sugars result in a more favorable Nutri-Score in a large variety of food groups. For instance, Nutri-Score may stimulate reformulation of various nutrients in composite dishes or cereals. However, as noted previously, the Nutri-Score's algorithm is based on a balance between “positive” and “negative” nutrients that may compensate for each other. For example, dairy drinks with added sugar that are low in saturated fat and salt may benefit from their naturally high protein content as this compensates for the sugar content, leading to a more favorable Nutri-Score (70). Also, adding extra protein to these beverages may make it seem they are reformulated, while their sugar content has not changed. Overall, monitoring food composition changes before and after introduction of Nutri-Score in European countries is crucial to evaluate the extent of producers' reformulation of products. As recommended in WHO's technical meeting on nutrient profiling (56), predictive validity would ideally be re-evaluated after adaptation of the algorithm. But more importantly, policy makers should be made aware that the predictive validity of a nutrient profile model is not a measure of effectiveness of a label using that model (56), and should be accompanied by an investigation of actual purchases of products with the label (68).

## Summary and conclusions

The Nutri-Score FOP label is one of the main candidates for standardized FOP labeling in the EU. The algorithm underpinning the Nutri-Score label is derived from FSA/Ofcom nutrient profile model, a model originally developed to regulate the marketing of foods to children in the UK. In line with WHO recommendations, content, convergent and predictive validity have been assessed in multiple studies (19–21, 23–29, 36–42), as reviewed here. However, their methodological approaches and conclusions on validity of the Nutri-Score should be interpreted with some caution. No gold standard for assessing healthfulness of products is available to date and this is not only problematic in the case of Nutri-Score, which is by far the most studied, but for the validation of many nutrient profile models currently existing. It must be noted that the large amount of articles that have been published on Nutri-Score does not necessarily mean that it is the best nutrient profile model. Content validity was based only on the distribution of the Nutri-Score categories in food groups, food categories and equivalent products of different brands. More insights into the actual products classified as having “higher” or “lower” nutritional quality is needed. Convergent validity was assessed by comparing the Nutri-Score classification with the French dietary guidelines, and adaptations were made to ensure alignment. This emphasizes the importance of taking national guidelines (12) into account while also limiting the generalisability of the validation process to other countries. Predictive validity was extensively assessed in the French context, with different adaptations of the Nutri-Score model. Yet, definite predictions on its effect on disease risk cannot yet be determined, as existing studies are not based on dietary patterns driven by Nutri-Score in particular.

Currently, seven countries are working on a joint Nutri-Score implementation (10). For some countries, content validity was evaluated (36–39), showing the ability of Nutri-Score to classify foods but not showing the difference in healthfulness of foods in different classes. Arguably, an evaluation of at least convergent validity within the context of these countries would be required, i.e., alignment with their respective dietary guidelines. Even if FOP labels and dietary guidelines serve different goals, they need to be aligned and provide a single coherent message to consumers. Failure to do so is likely to threaten the credibility and sustainability of both (58, 72). Ideally, predictive validity should be re-assessed once consensus is reached on adaptations in the algorithm. But even then, one should stay aware of the fact that the predictive validity of a nutrient profile model is not a measure of the effectiveness of a label using that model to improve diets (56). Therefore, besides validation of the algorithm itself, validation of its application and impact on purchases and dietary patterns in real life settings is crucial as well.

In conclusion, while Nutri-Score is one of the most studied FOP labels in Europe and its content, convergent and predictive validation have been extensively studied in the French context, more research is required on its validity and applicability within the European context. Will it be possible to adapt the algorithm in such way that it can be aligned with country-specific, food-based dietary guidelines and allows for product reformulation and innovation? Promisingly, an international committee was recently appointed to evaluate Nutri-Score, its underlying algorithm and its applicability in a European context. With this review, we aimed to provide a comprehensive evaluation of the validation process of the Nutri-Score algorithm to further the scientific and political process of nutrition labeling in the EU.

## Author contributions

ME and MR drafted the outline of the manuscript. DB and ME wrote the manuscript. MR, KG, and AR provided critical feedback and comments on the manuscript throughout all phases. All authors were involved in preparing this review paper and read and approved the final version of the manuscript.

## Funding

ME and MR were consultants to the Dutch Dairy Association and received financial support for conducting the literature search and writing the manuscript. The Dutch Dairy Association had no role in the design, analysis or writing of this review.

## Conflict of interest

ME and MR have called upon the Dutch government to make the introduction of the Nutri-Score label in the Netherlands conditional on alignment with national dietary guidelines, AR and KG supported this call. AR was a member of the international scientific committee of the Choices Programme. The remaining author declares that the research was conducted in the absence of any commercial or financial relationships that could be construed as a potential conflict of interest.

## Publisher's note

All claims expressed in this article are solely those of the authors and do not necessarily represent those of their affiliated organizations, or those of the publisher, the editors and the reviewers. Any product that may be evaluated in this article, or claim that may be made by its manufacturer, is not guaranteed or endorsed by the publisher.

## Abbreviations

BMI: Body Mass Index
CVD: Cardiovascular Disease
DI: Dietary Index
EU: European Union
FOP: Front-Of-Pack
FSA: Food Standards Agency
MTL: Multiple Traffic Light
NS: Nutri-Score
PNNS: Programme National Nutrition Santé
UK: United Kingdom
WHO: World Health Organisation.
