# Supplementary material for: The Nutri-Score algorithm: Evaluation of its validation process
Source: Front Nutr. 2022 Aug 15;9:974003. doi: 10.3389/fnut.2022.974003 (PMC9421047; doi:10.3389/fnut.2022.974003)
Supplement: Supplementary file 1 [file Data_Sheet_1.docx]

**Supplementary files**

**Supplementary file S1. Selection of studies on content, convergent and predictive validity of the Nutri-Score algorithm**

The selection of studies for the present review was based on the overview of validation studies presented on the website of the [French Ministry of Public Health](https://solidarites-sante.gouv.fr/prevention-en-sante/preserver-sa-sante/nutrition/nutri-score/article/validation-de-l-algorithme-du-nutri-score-en-france) (lastly accessed Nov 6, 2020). As Nutri-Score, as front-of-pack label, was originally developed for France, we initially aimed to include only studies that addressed the validity of the Nutri-Score algorithm in the French context. However, given the developments on front-of-pack labelling in the EU, we additionally included validity studies in other European countries, also presented on the [website](https://solidarites-sante.gouv.fr/prevention-en-sante/preserver-sa-sante/nutrition/nutri-score/article/nutri-score-etudes-specifiques-pays-regions). To ensure completeness, an additional PubMed search was conducted. A total of 70 potentially relevant papers were retrieved using the following search terms (date of search: Nov 6, 2020):

(nutri-score [tiab] OR

front-of-pack* [tiab] OR

nutrient profil* [tiab]) AND

(French [tiab] OR

France [tiab])

* limited to English

Abstracts of studies were read and based on these abstracts, a selection of studies was made. A total of 16 relevant validation studies, focussing on either content, convergent or predictive validity of the algorithm underpinning Nutri-Score, were selected from the website. Another two studies were included as they focused on the validation of the algorithm-based dietary index that was used in the predictive validity studies. Abstracts of additional potentially relevant papers found in PubMed were read, from this no additional papers were selected.

Selected studies (France)

*Content validity:*

- Julia C, Kesse-Guyot E, Touvier M, Méjean C, Fezeu L, Hercberg S. Application of the British Food Standards Agency nutrient profiling system in a French food composition database. Br J Nutr 2014;112(10):1699-1705.
- Julia C, Kesse-Guyot E, Ducrot P, Péneau S, Touvier M, Méjean C, Hercberg S. Performance of a five category front-of-pack labelling system–the 5-colour nutrition label–to differentiate nutritional quality of breakfast cereals in France. BMC Public Health 2015;15(1):179.

*Content and convergent validity:*

- Julia C, Ducrot P, Péneau S, et al. Discriminating nutritional quality of foods using the 5-Color nutrition label in the French food market: consistency with nutritional recommendations. Nutr J 2015;14(1):100.

*Predictive validity including validity studies dietary index:*

- Julia C, Touvier M, Méjean C, Ducrot P, Péneau S, Hercberg S, Kesse-Guyot E. Development and validation of an individual dietary index based on the British Food Standard Agency nutrient profiling system in a French context. J Nutr 2014;144(12):2009-2017.
- Julia C, Méjean C, Touvier M, et al. Validation of the FSA nutrient profiling system dietary index in French adults—findings from SUVIMAX study. Eur J Nutr 2016;55(5):1901-1910.
- Julia C, Fezeu L, Ducrot P, et al. The nutrient profile of foods consumed using the British Food Standards Agency nutrient profiling system is associated with metabolic syndrome in the SU. VI. MAX Cohort. J Nutr 2015;145(10):2355-2361.
- Julia C, Ducrot P, Lassale C, et al. Prospective associations between a dietary index based on the British Food Standard Agency nutrient profiling system and 13-year weight gain in the SU. VI. MAX cohort. Prev Med 2015;81:189-194*.*
- Donnenfeld M, Julia C, Kesse-Guyot E, et al. Prospective association between cancer risk and an individual dietary index based on the British Food Standards Agency nutrient profiling system. Br J Nutr 2015;114(10):1702-1710.
- Adriouch S, Julia C, Kesse-Guyot E, et al. Prospective association between a dietary quality index based on a nutrient profiling system and cardiovascular disease risk. Eur J Prev Cardiol 2016;23(15):1669-1676.
- Adriouch S, Julia C, Kesse-Guyot E, et al. Association between a dietary quality index based on the food standard agency nutrient profiling system and cardiovascular disease risk among French adults. Int J Cardiol 2017;234:22-27.
- Deschasaux M, Julia C, Kesse-Guyot E, et al. Are self-reported unhealthy food choices associated with an increased risk of breast cancer ? Prospective cohort study using the British Food Standards Agency nutrient profiling system. BMJ Open 2017;7(6):eo13718.
- Egnell M, Seconda L, Neal B, et al. Prospective associations of the original Food Standards Agency nutrient profiling system and three variants with weight gain, overweight and obesity risk: results from the French NutriNet-Santé cohort. Br J Nutr 2020; doi.10.1017/S0007114520003384.

Selected studies (European)

*Content validity*

- Dréano-Trecant L, Egnell M, Hercberg S, et al. Performance of the front-of-pack nutrition label Nutri-Score to discriminate the nutritional quality of products: a comparative study across 8 European countries. Nutrient 2020;12(5):1303.
- Szabo de Edelenyi F, Egnell M, Galan P, Druesne-Pecollo N, Hercberg S, Julia C. Ability of the Nutri-Score front-of-pack nutrition label to discriminate the nutritional quality of foods in the German food market and consistency with nutritional recommendations. Arch Publ Health 2019;77:28. doi:10.1186/s13690-019-0357-x.
- Szabo de Edelenyi F, Egnell M, Galan P, Hercberg S, Julia C. Rapport Technique de l’Equipe de Recherche en Epidémiologie Nutritionnelle – Ability of the front-of-pack nutrition label Nutri-Score to discriminate nutritional quality of food products in 7 European countries (Spain, Switzerland, Belgium, Italy, UK, Netherlands and Sweden) and consistency with nutritional recommendations. July 2019, available online at [www.nutriscore.blog](http://www.nutriscore.blog) (accessed 20-11-2020).

*Predictive validity:*

- Deschasaux M, Huybrechts I, Murphy N, et al. Nutritional quality of food as represented by the FSAm-NPS nutrient profiling system underlying the Nutri-Score label and cancer risk in Europe: Results from the EPIC prospective cohort study. Plos Med 2018;15(9):e1002651.
- Deschasaux M, Huybrechts I, Julia C, et al. Association between nutritional profiles of foods underlying Nutri-Score front-of-pack labels and mortality: EPIC cohort study in 10 European countries. BMJ 2020;370:m3173. doi:10.1136/bmj.m3173.
- Gómez-Donoso C, Martínez-González MA, Pérez-Cornago A, et al. Association between the nutrient profile system underpinning the Nutri-Score front-of-pack nutrition label and mortality in the SUN project: a prospective cohort study. Clin Nutr 2020. doi: 10.1016/j.clnu2020.07.008.

**Supplementary file S2. Overview of algorithms used in the Nutri-Score algorithm validation studies**

*FSA/Ofcom algorithm*

In the FSA/Ofcom algorithm, points are allocated according to the nutrient content for 100 g of foods or beverages. N- (negative) points are points allocated for ‘negative’ nutrients, P- (positive) points are points for ‘positive’ nutrients. (Note: in the original FSA/Ofcom algorithm, N-points are referred to as A-points and P-points as C-points; however for clarity, we prefer the terms N-points and P-points).

| **N-points** | Energy (kJ) | Saturated fat (g) | Total sugar (g) | Sodium (mg) |
| --- | --- | --- | --- | --- |
| 0 | ≤ 335 | ≤ 1 | ≤ 4.5 | ≤ 90 |
| 1 | > 335 | > 1 | > 4.5 | > 90 |
| 2 | > 670 | > 2 | > 9 | > 180 |
| 3 | > 1005 | > 3 | > 13.5 | > 270 |
| 4 | > 1340 | > 4 | > 18 | > 360 |
| 5 | > 1675 | > 5 | > 22.5 | > 450 |
| 6 | > 2010 | > 6 | > 27 | > 540 |
| 7 | > 2345 | > 7 | > 31 | > 630 |
| 8 | > 2680 | > 8 | > 36 | > 720 |
| 9 | > 3015 | > 9 | > 40 | > 810 |
| 10 | > 3350 | > 10 | > 45 | > 900 |

| **P-points** | Fruit, vegetables, legumes & nuts (%) | Fibre (non-starch polysaccharides) (g) | Protein (g) |
| --- | --- | --- | --- |
| 0 | ≤ 40 | ≤ 0.7 | ≤ 1.6 |
| 1 | > 40 | >0.7 | > 1.6 |
| 2 | > 60 | > 1.4 | > 3.2 |
| 3 | - | > 2.1 | > 4.8 |
| 4 | - | > 2.8 | > 6.4 |
| 5 | > 80 | > 3.5 | > 8.0 |

Total N-points = (points for energy) + (points for saturated fat) + (points for total sugar) + (points for sodium)

Total P-points = (points for fruits, vegetables, legumes & nuts [FVLN]) + (points for fibre) + (points for protein)

*Calculation FSA-score:*

* if N-points < 11; FSA-score = N-points – P-points

* if N-points ≥ 11 & points FVLN = 5; FSA score = N-points – P-points

* if N-points ≥ 11 & points FVLN < 5; FSA score = N-points – (points FVLN + points fibre)

*The proposed Nutri-Score algorithm*

In their paper, Julia *et al.* (2015)^(21)^ proposed changes to the FSA-algorithm for fats, beverages, and cheese. These changes were suggested to better rank according to nutrient profile, consistently with nutritional recommendations. In addition, the computation of the content of FVLN was modified:

*1) Points allocation for fats*

Adaptation of the points for saturated fats; starting at 6 g/100 g, after which an ascending step of 1 point is attributed per each 4 g/100 g saturated fats.

| **Points** | Saturated fat (g) |
| --- | --- |
| 0 | < 6 |
| 1 | ≥ 6 |
| 2 | ≥ 10 |
| 3 | ≥ 14 |
| 4 | ≥ 18 |
| 5 | ≥ 22 |
| 6 | ≥ 26 |
| 7 | ≥ 30 |
| 8 | ≥ 34 |
| 9 | ≥ 38 |
| 10 | ≥ 42 |

*2) Points allocation for beverages*

- Adaptation of points allocation for energy, starting at 0 kJ/100 g with an ascending step of 1 point attributed per 30 kJ/100 g g/100 g;

- Adaptation of points allocation for total sugar, starting at 0 g/ 100 g with an ascending step of 1 point attributed per 1,5 g/ 100 g, and taking into account the presence of sweeteners.

| **Points** | Energy (kJ) | Total sugar (g) |
| --- | --- | --- |
| 0 | ≤ 0 | ≤ 0 except if sweeteners |
| 1 | ≤ 30 | ≤ 1.5 or if sweeteners |
| 2 | ≤ 60 | ≤ 3 |
| 3 | ≤ 90 | ≤ 4.5 |
| 4 | ≤ 120 | ≤ 6 |
| 5 | ≤ 150 | ≤ 7.5 |
| 6 | ≤ 180 | ≤ 9 |
| 7 | ≤210 | ≤ 10.5 |
| 8 | ≤ 240 | ≤ 12 |
| 9 | ≤ 270 | ≤ 13.5 |
| 10 | > 270 | > 13.5 |

*3) Computation of FVLN content for all foods and beverages*

Adaptation to exclude dried fruits and oleaginous fruits, so that % FVLN is based only on all other fruits, vegetables and legumes.

*4) Score computation for cheese*

Adaptation in the score computation to take into account protein content, irrespective of total N-points:

FSA-score_cheese_ = N-points – P-points

*The Nutri-Score algorithm as defined by the French High Council for Public Health per June 2015*

In the report of the French High Council for Public Health^3^, further adaptations were made to the algorithm, based on additional research of the French National Agency for Food, Environmental and Occupational Health & Safety into the classification of products. The proposed adaptation for content of FVLN was not adopted, the proposed adaptation for cheese was. For saturated fats and beverages, the adaptations were different from those proposed by Julia *et al.* (2015)^(21)^. The adaptations that were effective as of June 2015, were:

*1) Points allocation for fats*

Adaptation of the points for saturated fats; starting at 10% saturated fats of total lipids, with an ascending step of 1 point attributed per each 6% increase.

| **Points** | Saturated fat/Lipids (%) |
| --- | --- |
| 0 | < 10 |
| 1 | < 16 |
| 2 | < 22 |
| 3 | < 28 |
| 4 | < 34 |
| 5 | < 40 |
| 6 | < 46 |
| 7 | < 52 |
| 8 | < 58 |
| 9 | < 64 |
| 10 | ≥ 64 |

*2) Points allocation for beverages*

- Adaptation of points allocation for energy, starting at 0 kJ/100 g with an ascending step of 1 point attributed

per 30 kJ/100;

- Adaptation of points allocation for total sugar, starting at 0 g/ 100 g with an ascending step of 1 point attributed per 1,5 g/ 100 g, and taking into account the presence of sweeteners;

- Adaption of points allocation for content of FVLN, and a corresponding change in score computation, increasing FVLN points from 5 to 10.

| **Points** | Energy (kJ) | Total sugar (g) | FVLN (%) |
| --- | --- | --- | --- |
| 0 | ≤ 0 | ≤ 0 except if sweeteners | < 40 |
| 1 | ≤ 30 | ≤ 1.5 or if sweeteners |  |
| 2 | ≤ 60 | ≤ 3 | > 40 |
| 3 | ≤ 90 | ≤ 4.5 |  |
| 4 | ≤ 120 | ≤ 6 | > 60 |
| 5 | ≤ 150 | ≤ 7.5 |  |
| 6 | ≤ 180 | ≤ 9 |  |
| 7 | ≤210 | ≤ 10.5 |  |
| 8 | ≤ 240 | ≤ 12 |  |
| 9 | ≤ 270 | ≤ 13.5 |  |
| 10 | > 270 | > 13.5 | > 80 |

FSA-score_beverages_ = N-points – P-points; if N-points < 11 or if N-points ≥ 11 & points FVLN = 10

FSA-score_beverages_ = N-points – (FVLN + fibre); if N-points ≥ 11 & points FVLN < 10

*3) Score computation for cheese*

Adaptation in the score computation to take into account protein content, irrespective of total N-points:

FSA-score_cheese_ = N-points – P-points

*The current Nutri-Score algorithm*

The current Nutri-Score algorithm uses the AOAC-thresholds to define fibre content of a product, rather than the NSP-thresholds as used in the validation studies. It also uses 100 ml as measurement unit for liquids, in line with the nutrition declaration on the package to ensure transparency to the consumer. Also, in October 2019, further adaptations were made to the algorithm to be able to better take into account the nutritional recommendations for oils in Europe. For this, the computation of the content for FVLN was adapted to include also the content of rapeseed oil, walnut oil and olive oil. For reference and details, see: <https://www.santepubliquefrance.fr/content/download/150263/file/QR_scientifique_technique_EN_12052020.pdf>

*
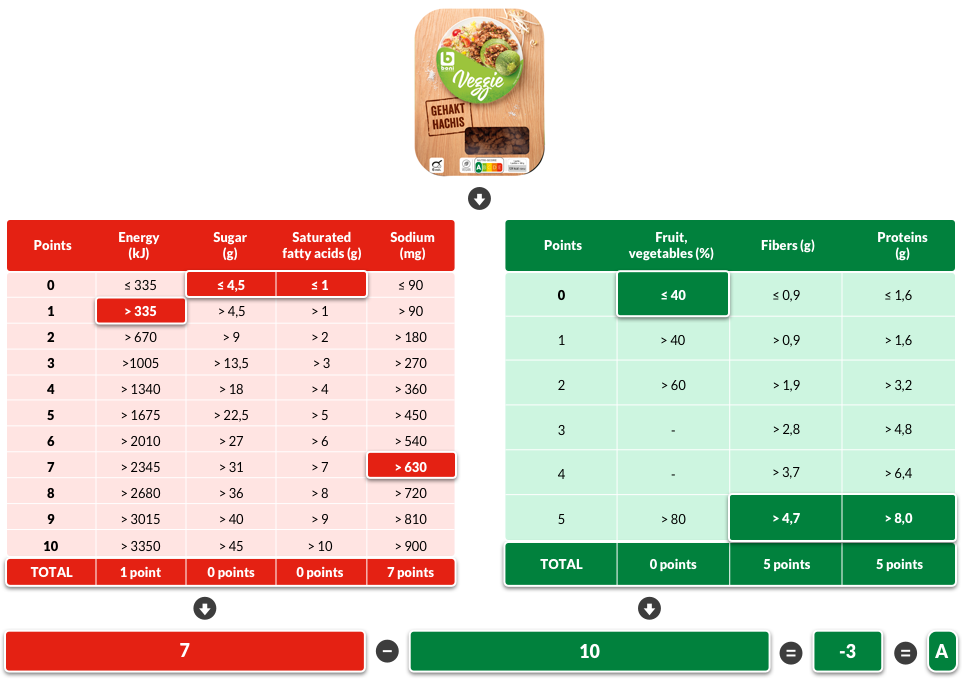
An example: calculation of the Nutri-Score (using the current algorithm) for vegan alternative for minced meat*

| **N-points** | Energy (kJ) | Saturated fat (g) | Total sugar (g) | Sodium (mg) |  | **P-points** | Fruit, vegetables, legumes & nuts (%) | Fibre (g) | Protein (g) |
| --- | --- | --- | --- | --- | --- | --- | --- | --- | --- |
| **0** | ≤ 335 | **< 1** | **≤ 4.5** | ≤ 90 |  | **0** | **≤ 40** | ≤ 0.9 | ≤ 1.6 |
| **1** | **> 335** | > 1 | > 4.5 | > 90 |  | **1** | > 40 | >0.9 | > 1.6 |
| **2** | > 670 | > 2 | > 9 | > 180 |  | **2** | > 60 | > 1.9 | > 3.2 |
| **3** | > 1005 | > 3 | > 13.5 | > 270 |  | **3** | - | > 2.8 | > 4.8 |
| **4** | > 1340 | > 4 | > 18 | > 360 |  | **4** | - | > 3.7 | > 6.4 |
| **5** | > 1675 | > 5 | > 22.5 | > 450 |  | **5** | > 80 | **> 4.7** | **> 8.0** |
| **6** | > 2010 | > 6 | > 27 | > 540 |  |  |  |  |  |
| **7** | > 2345 | > 7 | > 31 | **> 630** |  | **total points** | 0 | 5 | 5 |
| **8** | > 2680 | > 8 | > 36 | > 720 |  |  |  |  |  |
| **9** | > 3015 | > 9 | > 40 | > 810 |  |  |  |  |  |
| **10** | > 3350 | > 10 | > 45 | > 900 |  |  |  |  |  |
|  |  |  |  |  |  |  |  |  |  |
| **total points** | 1 | 0 | 0 | 7 |  |  |  |  |  |

Total N-points = 8

Total P-Points = 10

Nutri-Score = 8 – 10 = -2 = A

**Supplementary file S3. Distribution of food groups, food categories and beverage categories across quintiles (for foods) and quartiles (for beverages) of the FSA-score distribution, as presented in Julia *et al.* (2014)^(19)^ and Julia *et al.* (2015)^(21)^**

*Table A. Results from Julia et al. (2014)^(19)^: Distribution (%)^a^ of food groups and food categories across quintiles of FSA-score distribution in the French NutriNet Santé food composition database (non-weighted analyses, n=3,331)*

| **Food group** | | **Q1 (<-2)** | **Q2 (-1;3)** | **Q3 (4;11)** | **Q4 (12;16)** | **Q5 (≥17)** | **n** |
| --- | --- | --- | --- | --- | --- | --- | --- |
| Fruits & vegetables | | 66.2 | 22.9 | 10.3 | 0.6 | 0.0 | 340 |
|  | Fruits | 70.4 | 14.1 | 15.6 | 0.0 | 0.0 | 135 |
|  | Dried Fruits | 16.7 | 55.6 | 16.7 | 11.1 | 0.0 | 18 |
|  | Vegetables | 93.7 | 4.8 | 1.6 | 0.0 | 0.0 | 126 |
|  | Soups | 14.8 | 70.5 | 14.8 | 0.0 | 0.0 | 61 |
|  | |  |  |  |  |  |  |
| Cereals, legumes & potatoes | | 29.3 | 20.4 | 30.5 | 15.1 | 4.7 | 338 |
|  | Cereals | 60.0 | 35.0 | 3.3 | 1.7 | 0.0 | 60 |
|  | Breakfast cereals | 7.5 | 4.1 | 46.9 | 30.6 | 10.9 | 147 |
|  | Legumes | 100.0 | 0.0 | 0.0 | 0.0 | 0.0 | 14 |
|  | Bread | 27.4 | 36.9 | 29.8 | 6.0 | 0.0 | 84 |
|  | Potatoes | 45.5 | 33.3 | 21.2 | 0.0 | 0.0 | 33 |
|  | |  |  |  |  |  |  |
| Milk & dairy products | | 4.4 | 25.6 | 28.4 | 18.0 | 23.6 | 550 |
|  | Dairy desserts | 3.9 | 33.0 | 47.7 | 14.0 | 1.6 | 258 |
|  | Cheese | 0.6 | 4.9 | 6.7 | 16.5 | 71.3 | 164 |
|  | Ice cream | 1.6 | 3.2 | 24.2 | 56.5 | 14.5 | 62 |
|  | Milk and yogurt | 18.2 | 69.7 | 10.6 | 1.5 | 0.0 | 66 |
|  | |  |  |  |  |  |  |
| Meat, fish & eggs | | 27.9 | 32.5 | 11.5 | 11.8 | 16.4 | 391 |
|  | Offals | 30.4 | 39.1 | 13.0 | 8.7 | 8.7 | 46 |
|  | Processed meat | 2.4 | 4.7 | 9.4 | 18.8 | 64.7 | 85 |
|  | Eggs | 11.8 | 41.2 | 11.8 | 23.5 | 11.8 | 17 |
|  | Fish and seafood | 46.0 | 33.3 | 8.7 | 9.5 | 2.4 | 126 |
|  | Meat | 28.2 | 47.9 | 15.4 | 8.6 | 0.0 | 117 |
|  | |  |  |  |  |  |  |
| Sugary snacks | | 1.1 | 3.0 | 17.1 | 31.5 | 47.2 | 794 |
|  | Biscuits and cakes | 0.2 | 3.8 | 20.6 | 32.9 | 42.6 | 423 |
|  | Chocolate products | 1.4 | 0.9 | 6.9 | 11.5 | 79.3 | 217 |
|  | Sweets | 4.5 | 3.6 | 23.2 | 55.4 | 13.4 | 112 |
|  | Pastries | 0.0 | 4.8 | 19.0 | 57.1 | 19.1 | 42 |
|  | |  |  |  |  |  |  |
| Salty snacks | | 15.5 | 16.2 | 31.1 | 18.2 | 18.9 | 148 |
|  | Nuts | 42.0 | 34.0 | 24.0 | 0.0 | 0.0 | 50 |
|  | Appetizers | 2.0 | 7.1 | 34.7 | 27.6 | 28.6 | 98 |
|  | |  |  |  |  |  |  |
| Fats & sauces | | 2.9 | 9.6 | 19.9 | 20.6 | 47.1 | 136 |
|  | Fats | 0.0 | 4.9 | 4.9 | 23.0 | 67.2 | 61 |
|  | Dressings and sauces | 5.3 | 13.3 | 32.-0 | 18.7 | 30.7 | 75 |
|  | |  |  |  |  |  |  |
| Composite dishes | | 19.1 | 34.7 | 20.7 | 18.9 | 6.6 | 634 |
|  | Pizza, pies and quiche | 1.0 | 19.1 | 29.5 | 38.1 | 12.4 | 105 |
|  | One-dish meals | 26.7 | 40.7 | 19.3 | 10.3 | 2.9 | 445 |
|  | Sandwich | 1.2 | 22.6 | 16.7 | 40.5 | 19.1 | 84 |

^a^ percentages reported were rounded to the nearest decimal for the present review

*Table B. Results from Julia et al. (2014)^(19)^: Distribution (%)^a^ of beverage categories across quartiles of FSA-score distribution in the French NutriNet Santé food composition database (non-weighted analyses, n=177)*

| **Beverage category** | | **Q1 (<-1)** | **Q2 (0)** | **Q3 (1)** | **Q4 (≥2)** | **n** |
| --- | --- | --- | --- | --- | --- | --- |
|  | Artificially sweetened | 8.7 | 91.3 | 0 | 0 | 23 |
|  | Non-sugared | 12.8 | 69.2 | 7.7 | 10.3 | 39 |
|  | Sweetened | 6.7 | 5.3 | 32 | 56 | 75 |
|  | Fruit juice | 90.6 | 6.3 | 0 | 3.1 | 32 |
|  | Fruit nectar | 12.5 | 25 | 12.5 | 50 | 8 |

^a^ percentages reported were rounded to the nearest decimal for the present review

*Table C. Results from Julia et al. (2015)^(21)^: Distribution (%) of food groups and food categories across quintiles of FSA-score distribution in the Open Food Facts database (n=7,777)*

| **Food group** | | **Q1 (<-2)** | **Q2 (-1;3)** | **Q3 (4;11)** | **Q4 (12;16)** | **Q5 (≥17)** | **N** |
| --- | --- | --- | --- | --- | --- | --- | --- |
| Fruits & vegetables | | 72.1 | 23.3 | 4.3 | 0.4 | - | 748 |
|  | Vegetables | 87.7 | 10.6 | 1.2 | 0.5 | - | 405 |
|  | Dried Fruits | 18.2 | 66.7 | 12.1 | 3.0 | - | 33 |
|  | Fruits | 94.5 | 3.8 | 1.6 | - |  | 182 |
|  | Soups | 5.8 | 77.7 | 16.5 | - |  | 103 |
| Cereals, legumes & potatoes | | 51.7 | 20.8 | 19.6 | 6.4 | 1.5 | 1335 |
|  | Bread | 32 | 37.5 | 24.9 | 4.2 | 1.3 | 309 |
|  | Pasta, rice & cereals | 78.6 | 17.4 | 3.8 | 0.2 | - | 552 |
|  | Legumes | 99.1 | - | 0.9 | - |  | 106 |
|  | Potatoes | 41.1 | 44.4 | 11.1 | 3.3 | - | 90 |
|  | Breakfast cereals | 5.4 | 9.4 | 54.7 | 24.8 | 5.8 | 278 |
| Meat, fish & eggs | | 5.1 | 37.9 | 26.9 | 12.9 | 17.2 | 791 |
|  | Eggs | - | 100 | - | - | - | 33 |
|  | Fis & seafood | 9.5 | 49.0 | 28.1 | 13.1 | 0.3 | 306 |
|  | Meat | 7.8 | 50.8 | 29.7 | 8.6 | 3.1 | 128 |
|  | Offals | 6.7 | 13.3 | 13.3 | 60.0 | 6.7 | 15 |
|  | Processed meat | - | 16.2 | 28.2 | 13.6 | 42.1 | 309 |
| Milk & dairy products | | 5.2 | 34.1 | 20.9 | 15.8 | 24.0 | 928 |
|  | Milk and yogurt | 10.9 | 64.8 | 15.8 | 6.9 | 1.7 | 423 |
|  | Cheese | - | 3.5 | 1.2 | 22 | 73.3 | 255 |
|  | Dairy desserts | 1.5 | 16.9 | 55.9 | 23.5 | 2.2 | 136 |
|  | Ice cream | - | 8.8 | 42.1 | 26.3 | 22.8 | 114 |
| Fats & sauces | | 2.2 | 15.6 | 19.1 | 24.9 | 38.2 | 461 |
|  | Dressings & sauces | 3.7 | 26.1 | 30.9 | 26.8 | 12.5 | 272 |
|  | Fats | - | 0.5 | 2.1 | 22.2 | 75.1 | 189 |
| Salty snacks | | 2.9 | 9.8 | 45.0 | 25.6 | 16.7 | 480 |
|  | Appetizers | 0.6 | 3.7 | 47.7 | 29.1 | 18.9 | 323 |
|  | Nuts | 15.5 | 29.3 | 50.0 | 5.2 | - | 58 |
|  | Salty & fatty products | 3.0 | 18.2 | 33.3 | 26.3 | 19.2 | 99 |
| Sugary snacks | | 0.5 | 1.8 | 11.7 | 25.2 | 60.8 | 1467 |
|  | Biscuits and cakes | 0.5 | 1.5 | 8.5 | 26.8 | 62.8 | 792 |
|  | Chocolate products | - | 0.5 | 9.0 | 8.5 | 82.0 | 378 |
|  | Pastries | 1.1 | 4.6 | 17.2 | 47.1 | 29.9 | 87 |
|  | Sweets | 1.0 | 4.3 | 26.7 | 40 | 28.1 | 210 |
| Composite foods | | 19.8 | 49.4 | 20.7 | 7.9 | 2.3 | 774 |
|  | One-dish meals | 20.4 | 54.1 | 19.4 | 4.7 | 1.3 | 593 |
|  | Pizza, pies and quiche | 2.6 | 27.3 | 41.6 | 23.4 | 5.2 | 77 |
|  | Sandwich | 11.5 | 26.9 | 21.2 | 28.8 | 11.5 | 52 |
|  | Side dishes | 46.2 | 50 | 3.8 | - |  | 52 |

*Table D. Results from Julia et al. (2015)^(21)^: Distribution (%) of beverage categories across quartiles of FSA-score distribution in the Open Food Facts database (n=793)*

| **Beverage category** | | **Q1 (<-1)** | **Q2 (0)** | **Q3 (1)** | **Q4 (≥2)** | **n** |
| --- | --- | --- | --- | --- | --- | --- |
|  | Water and flavoured water | - | 100 | - | - | 20 |
|  | Tea, herbal tea and coffee | - | 100 | - | - | 55 |
|  | Fruit juice | 99.3 | - | 0.3 | 0.3 | 286 |
|  | Fruit nectar | - | - | 17.6 | 82.4 | 34 |
|  | Fruit flavoured still drinks | 19.2 | 7.7 | 38.5 | 34.6 | 78 |
|  | Artificially sweetened | 1.3 | 88.8 | 6.3 | 3.8 | 80 |
|  | Sweetened | 0.4 | 5.8 | 34.2 | 59.6 | 240 |

**Supplementary file S4. Country-specific distributions (%)^a^ of food groups^b^ across Nutri-Score classes, as reported for Switzerland, Sweden, Slovakia, Portugal, Poland, Norway, France and Finland in Dréano-Trécant *et al.* (2020)^(37)^**

| **Food group** | | **A** | **B** | **C** | **D** | **E** | **n** |
| --- | --- | --- | --- | --- | --- | --- | --- |
| Egg or egg product | |  |  |  |  |  |  |
|  | Switzerland | 66.7 | - | - | 33.3 | - | 3 |
|  | Sweden | 55.6 | 11.1 | 11.1 | 22.2 | - | 9 |
|  | Slovakia | 66.7 | 6.7 | 6.7 | 20.0 | - | 15 |
|  | Portugal | 45.5 | 9.1 | 36.4 | 9.1 | - | 11 |
|  | Poland | 50.0 | - | - | 25.0 | 25.0 | 4 |
|  | Norway | 50.0 | 20.0 | 30.0 | - | - | 10 |
|  | France | 33.3 | 33.3 | - | 20.0 | 13.3 | 15 |
|  | Finland | 62.5 | 12.5 | 12.5 | 12.5 | - | 8 |
| Fat or oil | |  |  |  |  |  |  |
|  | Switzerland | - | - | 33.3 | 59.3 | 7.4 | 27 |
|  | Sweden | - | - | 15.4 | 66.2 | 18.5 | 65 |
|  | Slovakia | - | - | 14.3 | 71.4 | 14.3 | 28 |
|  | Portugal | - | - | 11.1 | 63.0 | 25.9 | 27 |
|  | Poland | - | - | 33.3 | 62.5 | 4.2 | 24 |
|  | Norway | - | - | 8.3 | 63.9 | 27.8 | 36 |
|  | France | - | - | 18.5 | 69.2 | 12.3 | 65 |
|  | Finland | - | - | 15.6 | 54.7 | 29.7 | 64 |
|  |  |  |  |  |  |  |  |
| Fruit or fruit product | |  |  |  |  |  |  |
|  | Switzerland | 7.7 | 20.7 | 8.6 | - | - | 58 |
|  | Sweden | 67.7 | 21.5 | 10.8 | - | - | 93 |
|  | Slovakia | 61.4 | 26.1 | 12.5 | - | - | 88 |
|  | Portugal | 71.4 | 6.4 | 22.2 | - | - | 63 |
|  | Poland | 74.1 | 18.5 | 7.4 | - | - | 54 |
|  | Norway | 75.0 | 22.1 | 2.9 | - | - | 68 |
|  | France | 81.1 | 14.9 | 4.1 | - | - | 74 |
|  | Finland | 88.0 | 9.3 | 2.7 | - | - | 75 |
| Grain or grain product | |  |  |  |  |  |  |
|  | Switzerland | 29.4 | 9.3 | 17.0 | 27.8 | 16.5 | 194 |
|  | Sweden | 37.3 | 15.8 | 17.2 | 19.1 | 10.6 | 303 |
|  | Slovakia | 25.4 | 14.8 | 23.9 | 29.6 | 6.3 | 142 |
|  | Portugal | 34.0 | 14.9 | 14.9 | 27.7 | 8.5 | 94 |
|  | Poland | 32.7 | 19.1 | 14.8 | 25.3 | 8.0 | 162 |
|  | Norway | 41.9 | 11.6 | 14.5 | 23.7 | 8.3 | 241 |
|  | France | 22.4 | 4.8 | 19.0 | 36.8 | 17.0 | 353 |
|  | Finland | 31.1 | 33.7 | 17.1 | 11.0 | 7.1 | 409 |
|  |  |  |  |  |  |  |  |
| Meat or meat product | |  |  |  |  |  |  |
|  | Switzerland | 46.5 | 12.6 | 4.7 | 12.6 | 23.6 | 127 |
|  | Sweden | 33.9 | 13.4 | 12.4 | 25.2 | 14.1 | 298 |
|  | Slovakia | 56.3 | 14.7 | 8.4 | 20.0 | 0.5 | 190 |
|  | Portugal | 22.2 | 21.7 | 23.1 | 24.0 | 9.1 | 221 |
|  | Poland | 27.0 | 7.2 | 15.3 | 33.3 | 17.1 | 111 |
|  | Norway | 30.1 | 19.0 | 6.5 | 29.4 | 15.0 | 153 |
|  | France | 36.0 | 15.0 | 10.8 | 16.2 | 21.9 | 333 |
|  | Finland | 25.9 | 11.6 | 13.6 | 41.2 | 6.8 | 147 |
| Milk, milk product or milk substitute | |  |  |  |  |  |  |
|  | Switzerland | 16.1 | 19.6 | 13.4 | 43.8 | 7.1 | 112 |
|  | Sweden | 13.4 | 17.6 | 25.4 | 37.3 | 6.3 | 142 |
|  | Slovakia | 37.9 | 25.9 | 22.4 | 5.2 | 8.6 | 58 |
|  | Portugal | 20.6 | 32.9 | 8.2 | 23.3 | 15.1 | 73 |
|  | Poland | 18.2 | 28.8 | 16.7 | 27.3 | 9.1 | 66 |
|  | Norway | 10.8 | 15.7 | 14.7 | 44.1 | 14.7 | 102 |
|  | France | 12.6 | 15.6 | 12.1 | 54.3 | 5.5 | 199 |
|  | Finland | 17.5 | 31.8 | 21.4 | 24.7 | 4.6 | 154 |
| Composite food product | |  |  |  |  |  |  |
|  | Switzerland | 5.1 | 28.3 | 47.5 | 14.1 | 5.1 | 99 |
|  | Sweden | 18.1 | 28.9 | 29.2 | 20.48 | 3.4 | 537 |
|  | Slovakia | 3.6 | 26.3 | 38.1 | 22.1 | 10.0 | 281 |
|  | Portugal | 9.2 | 30.3 | 27.7 | 23.5 | 9.2 | 119 |
|  | Poland | 44.7 | 28.1 | 11.8 | 13.2 | 2.2 | 228 |
|  | Norway | 7.1 | 23.9 | 29.2 | 31.9 | 8.0 | 113 |
|  | France | 15.4 | 25.5 | 31.5 | 22.7 | 4.9 | 428 |
|  | Finland | 26.7 | 31.3 | 24.9 | 14.7 | 2.4 | 850 |
| Nut, seed or kernel | |  |  |  |  |  |  |
|  | Switzerland | 31.6 | 26.3 | 21.1 | 15.8 | 5.3 | 19 |
|  | Sweden | 28.0 | - | 60.0 | 12.0 | - | 25 |
|  | Slovakia | 28.1 | 21.9 | 40.6 | 9.4 | - | 32 |
|  | Portugal | 50.0 | 21.4 | 21.4 | 7.1 | - | 14 |
|  | Poland | 57.1 | 21.4 | - | 21.4 | - | 14 |
|  | Norway | 50.0 | 16.7 | 22.2 | 11.1 | - | 18 |
|  | France | 43.9 | 14.6 | 34.2 | 7.3 | - | 41 |
|  | Finland | 57.1 | 14.3 | 7.1 | 21.4 | - | 14 |
| Seafood or related product | |  |  |  |  |  |  |
|  | Switzerland | 65.6 | 15.6 | 6.3 | 9.4 | 3.1 | 32 |
|  | Sweden | 43.2 | 12.7 | 8.5 | 32.2 | 3.4 | 118 |
|  | Slovakia | 62.0 | 21.5 | 6.3 | 10.1 | - | 79 |
|  | Portugal | 47.6 | 21.8 | 11.1 | 9.5 | - | 126 |
|  | Poland | 50.0 | 17.7 | 11.8 | 20.6 | - | 34 |
|  | Norway | 26.6 | 27.8 | 18.9 | 22.5 | 4.1 | 169 |
|  | France | 68.3 | 16.3 | 7.5 | 7.1 | 0.9 | 227 |
|  | Finland | 43.9 | 10.6 | 13.6 | 31.8 | - | 66 |
| Sugar or sugar product | |  |  |  |  |  |  |
|  | Switzerland | - | 2.6 | 25.6 | 30.8 | 41.0 | 39 |
|  | Sweden | 2.5 | 9.2 | 31.7 | 35.0 | 21.7 | 120 |
|  | Slovakia | - | 2.8 | 13.9 | 79.2 | 4.2 | 72 |
|  | Portugal | - | 2.4 | 31.7 | 46.3 | 19.5 | 41 |
|  | Poland | 2.0 | - | 36.0 | 34.0 | 28.0 | 50 |
|  | Norway | - | 3.7 | 24.1 | 42.6 | 29.6 | 54 |
|  | France | 4.2 | 7.5 | 30.8 | 33.3 | 24.2 | 120 |
|  | Finland | 10.2 | 23.7 | 27.1 | 26.3 | 12.7 | 118 |
| Vegetable or vegetable product | |  |  |  |  |  |  |
|  | Switzerland | 98.5 | 1.5 | - | - | - | 66 |
|  | Sweden | 90.9 | 3.9 | 4.8 | 0.5 | - | 208 |
|  | Slovakia | 88.6 | 8.1 | 3.3 | - | - | 123 |
|  | Portugal | 92.5 | 2.2 | 5.4 | - | - | 93 |
|  | Poland | 96.2 | 2.9 | 1.0 | - | - | 104 |
|  | Norway | 95.1 | 2.0 | 2.9 | - | - | 102 |
|  | France | 95.3 | 3.1 | 1.6 | - | - | 256 |
|  | Finland | 87.8 | 5.1 | 7.1 | - | - | 98 |
| Beverage non-milk | |  |  |  |  |  |  |
|  | Switzerland | 37.0 | 9.6 | 20.6 | 9.6 | 23.3 | 73 |
|  | Sweden | 6.9 | 26.4 | 5.6 | 15.3 | 45.8 | 72 |
|  | Slovakia | 24.0 | 4.0 | 20.0 | 10.7 | 41.3 | 75 |
|  | Portugal | 12.8 | 23.1 | 18.0 | 7.7 | 38.5 | 39 |
|  | Poland | - | 19.1 | 33.8 | 4.4 | 42.7 | 68 |
|  | Norway | - | 17.1 | 11.4 | - | 71.4 | 35 |
|  | France | 45.5 | 11.1 | 11.6 | 11.6 | 20.2 | 198 |
|  | Finland | 5.6 | 29.2 | 23.6 | 13.9 | 27.8 | 72 |

^a^ Percentages rounded to the nearest decimal for the present review

^b^ Food composition data from the EUROFIR food database
